# *SHOOT MERISTEMLESS* participates in the heterophylly of *Hygrophila difformis* (Acanthaceae)

**DOI:** 10.1093/plphys/kiac382

**Published:** 2022-08-19

**Authors:** Gaojie Li, Jingjing Yang, Yimeng Chen, Xuyao Zhao, Yan Chen, Seisuke Kimura, Shiqi Hu, Hongwei Hou

**Affiliations:** The State Key Laboratory of Freshwater Ecology and Biotechnology, The Key Laboratory of Aquatic Biodiversity and Conservation of Chinese Academy of Sciences, Institute of Hydrobiology, Chinese Academy of Sciences, Wuhan 430072, China; The State Key Laboratory of Freshwater Ecology and Biotechnology, The Key Laboratory of Aquatic Biodiversity and Conservation of Chinese Academy of Sciences, Institute of Hydrobiology, Chinese Academy of Sciences, Wuhan 430072, China; The State Key Laboratory of Freshwater Ecology and Biotechnology, The Key Laboratory of Aquatic Biodiversity and Conservation of Chinese Academy of Sciences, Institute of Hydrobiology, Chinese Academy of Sciences, Wuhan 430072, China; College of Advanced Agricultural Sciences, University of Chinese Academy of Sciences, Beijing 100049, China; The State Key Laboratory of Freshwater Ecology and Biotechnology, The Key Laboratory of Aquatic Biodiversity and Conservation of Chinese Academy of Sciences, Institute of Hydrobiology, Chinese Academy of Sciences, Wuhan 430072, China; The State Key Laboratory of Freshwater Ecology and Biotechnology, The Key Laboratory of Aquatic Biodiversity and Conservation of Chinese Academy of Sciences, Institute of Hydrobiology, Chinese Academy of Sciences, Wuhan 430072, China; College of Advanced Agricultural Sciences, University of Chinese Academy of Sciences, Beijing 100049, China; Faculty of Life Sciences, Kyoto Sangyo University, Kyoto 603-8555, Japan; Center for Plant Sciences, Kyoto Sangyo University, Kyoto 603-8555, Japan; Zhejiang Marine Development Research Institute, Zhoushan 316021, China; The State Key Laboratory of Freshwater Ecology and Biotechnology, The Key Laboratory of Aquatic Biodiversity and Conservation of Chinese Academy of Sciences, Institute of Hydrobiology, Chinese Academy of Sciences, Wuhan 430072, China; College of Advanced Agricultural Sciences, University of Chinese Academy of Sciences, Beijing 100049, China

## Abstract

In heterophyllous plants, leaf shape shows remarkable plasticity in response to environmental conditions. However, transgenic studies of heterophylly are lacking and the molecular mechanism remains unclear. Here, we cloned the *KNOTTED1-LIKE HOMEOBOX* family gene *SHOOT MERISTEMLESS* (*STM*) from the heterophyllous plant *Hygrophila difformis* (Acanthaceae). We used molecular, morphogenetic, and biochemical tools to explore its functions in heterophylly. *HdSTM* was detected in different organs of *H. difformis*, and its expression changed with environmental conditions. Heterologous, ectopic expression of *HdSTM* in Arabidopsis (*Arabidopsis thaliana*) increased leaf complexity and *CUP-SHAPED COTYLEDON* (*CUC*) transcript levels. However, overexpression of *HdSTM* in *H. difformis* did not induce the drastic leaf change in the terrestrial condition. Overexpression of *HdSTM* in *H. difformis* induced quick leaf variations in submergence, while knockdown of *HdSTM* led to disturbed leaf development and weakened heterophylly in *H. difformis*. *HdCUC3* had the same spatiotemporal expression pattern as *HdSTM*. Biochemical analysis revealed a physical interaction between HdSTM and HdCUC3. Our results provide genetic evidence that *HdSTM* is involved in regulating heterophylly in *H. difformis*.

## Teaser

*SHOOT MERISTEMLESS* changes its expression pattern with the surrounding environment, regulating the heterophylly of *Hygrophila difformis*.

## Introduction

Plants show excellent leaf plasticity in response to environmental changes, known as heterophylly. These plants represent ideal model systems for studying plant adaptation to the environment (Zotz et al., 2011). Heterophylly has been widely observed in amphibious or aquatic plants and can be induced by diverse environmental factors (Kane and Albert, 1987; Sato et al., 2008; Nakayama et al., 2014; Li et al., 2019). In amphibious plants, the submerged leaves are generally deeply lobed, filiform or linear, thin, and lack stomata, whereas terrestrial leaves are simple and complete, with more stomata and vascular bundles (Koga et al., 2020; Li et al., 2021; van Veen and Sasidharan, 2021). Blue light efficiently induces terrestrial leaf formation in *Marsilea quadrifolia* under submerged conditions (Lin and Yang, 1999). In contrast, submerged leaf formation is caused by high light density or low temperature in *Rorippa aquatica* (Nakayama et al., 2014) and *Ludwigia arcuata* (Sato et al., 2008). Phytohormones are also critical regulators of heterophylly (Nakayama et al., 2017; Li et al., 2021). In *Ranunculus trichophyllus* and *L. arcuata*, abscisic acid (ABA) and ethylene play antagonistic roles in regulating leaf formation: Plants treated with ethylene form slender leaves with morphology similar to submerged leaves, whereas ABA induces the formation of broad leaves with morphology similar to terrestrial leaves (Kuwabara et al., 2003; Li et al., 2017; Kim et al., 2018). Gibberellic acid (GA) treatment promotes the formation of leaves with morphology similar to submerged leaves in *Callitriche heterophylla* (Deschamp and Cooke, 1984). In contrast, GA treatment induces the formation of terrestrial leaves in *R. aquatica* (Nakayama et al., 2014). These observations highlight the diverse responses of different plant species to environmental factors and phytohormones.

Leaf primordia initiate at the flanks of the shoot apical meristem (SAM). The functions of the SAM are maintained by class 1 *KNOTTED1-LIKE HOMEOBOX* (*KNOX1*) genes (Shani et al., 2006; Hay and Tsiantis, 2010; Moon and Hake, 2011). The *KNOX1* family genes *SHOOT MERISTEMLESS* (*STM*) and *BREVIPEDICELLUS* (*BP*) play key roles in SAM maintenance in Arabidopsis (*Arabidopsis thaliana*). *STM* is expressed in the SAM to maintain cells in an undifferentiated state, while *BP* plays redundant roles with *STM* (Long et al., 1996; Byrne et al., 2002; Shani et al., 2006; Scofield et al., 2013). *STM* is essential for plant development. It activates cytokinin (CK) biosynthesis and represses GA biosynthesis to maintain meristem activity; *stm* mutations result in a lack of SAM formation (Barton and Poethig, 1993; Jasinski et al., 2005). In addition, STM indirectly regulates the expression of *BP* through *ASYMMETRIC LEAVES1* (Byrne et al., 2002; Hay and Tsiantis, 2006; Rast-Somssich et al., 2015).

*CUP-SHAPED COTYLEDON* (*CUC*) genes and *STM* reinforce each other in many eudicots (Aida et al., 1999; Hibara et al., 2003; Spinelli et al., 2011). *CUC* genes encode NAM, ATAF1/2 and CUC2 (NAC) domain proteins that are highly similar to NO APICAL MERISTEM, which functions in cotyledon, organ boundary, and leaf margin development (Aida et al., 1997; Hibara et al., 2006; Nikovics et al., 2006; Blein et al., 2008; Kawamura et al., 2010). In the compound-leaf plant *Cardamine hirsuta*, *ChCUCs* are required for the leaflets formation, and the expression of *ChSTM* is strongly reduced in plants with downregulated *ChCUC* expression (Blein et al., 2008). *CUC1* and *CUC2* control leaf margin development in diverse species (Nikovics et al., 2006; Sha et al., 2018; Zheng et al., 2019), and *CUC3* is also essential for the initiation of the shoot and axillary meristems (Vroemen et al., 2003). Plants with silenced *ChCUC3* expression produced fewer and smoother leaflets than the wild-type (WT) (Blein et al., 2008). The continuous expression of *STM* in *A. thaliana* led to the activation of *CUC2* and *CUC3*, independently of *CUC1* (Spinelli et al., 2011). In heterophyllous plant *R. aquatica*, the expression of *STM* and *CUC3* changed with ambient surroundings. Both of them were upregulated in complex leaves (Nakayama et al., 2014), indicating their important functions in heterophylly.

**Figure 1:**
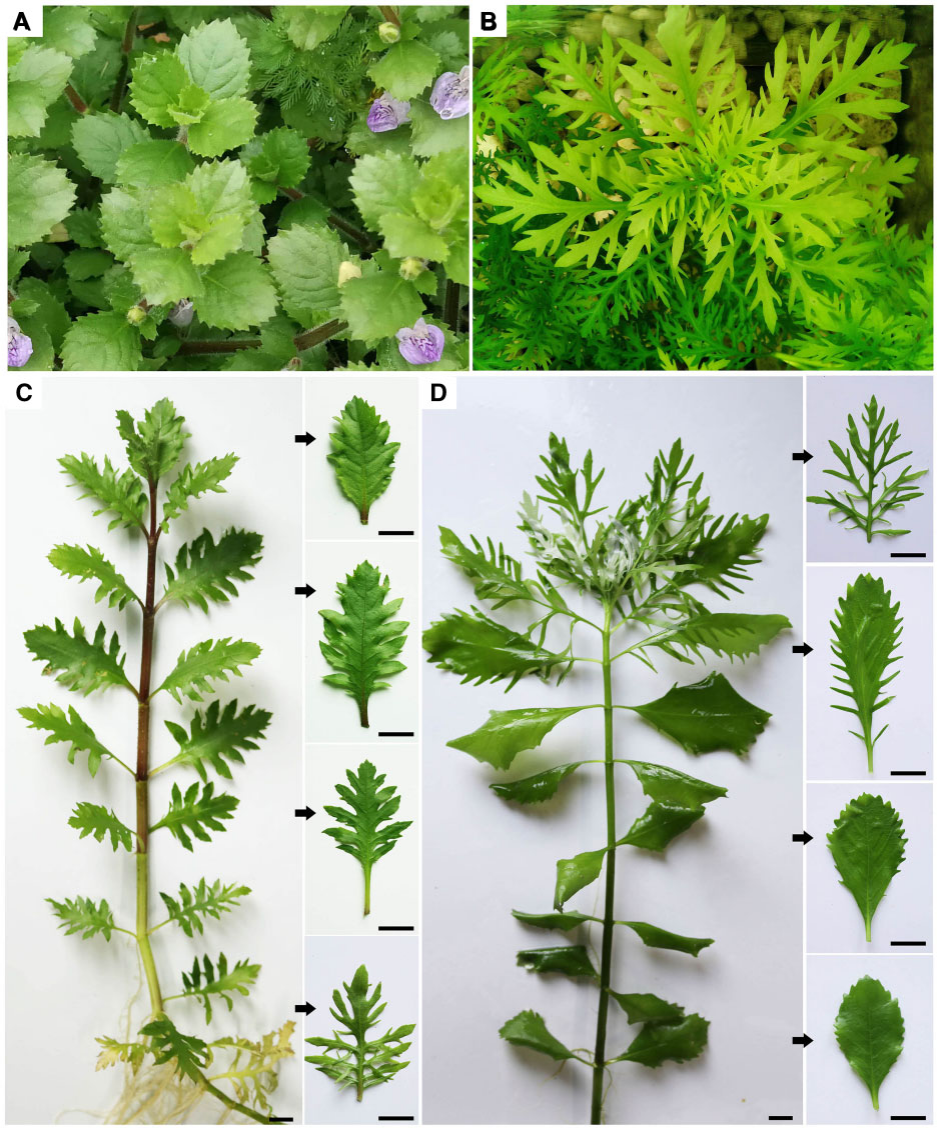
Phenotypes of *H. difformis* grown under different environmental conditions. A, Plants grown in a terrestrial environment. B, Plants grown in a submerged environment. C, A plant shifted from submerged to terrestrial conditions. Arrows indicate typical leaves in the process of acclimation to terrestrial conditions. D, A plant shifted from terrestrial to submerged conditions. Arrows indicate typical leaves in the process of acclimation to submerged conditions. Bars = 1 cm in (C–D).

The *KNOX1*–GA module functions in the regulation of heterophylly in *R. aquatica* (Nakayama et al., 2014). However, no transgenic studies of the roles of this module in heterophyllous species have been performed, and the underlying molecular mechanisms are still largely unknown (He et al., 2018; Kim et al., 2018; Li et al., 2021). *Hygrophila difformis* (Acanthaceae), a semi-aquatic plant sensitive to environmental factors, has a variety of leaf shapes, from simple serrated leaves to highly complex leaves, under different conditions (Figure 1). In addition, a system for *Agrobacterium tumefaciens*-mediated transformation of this plant was recently developed (Li et al., 2020). Therefore, *H. difformis* represents an ideal system to study the molecular mechanisms underlying heterophylly (Li et al., 2017, 2021).

In this study, we cloned the *STM* ortholog *HdSTM* from *H. difformis*. We showed that *HdSTM* is involved in the heterophylly of *H. difformis* by molecular, morphogenetic, and biochemical methods. Our findings shed light on the *HdSTM* regulating heterophylly in *H. difformis* and the molecular mechanisms regulating the development of above-ground organs in eudicot plants.

## Results

### Isolation of *HdSTM* in *H. difformis*

**Figure 2:**
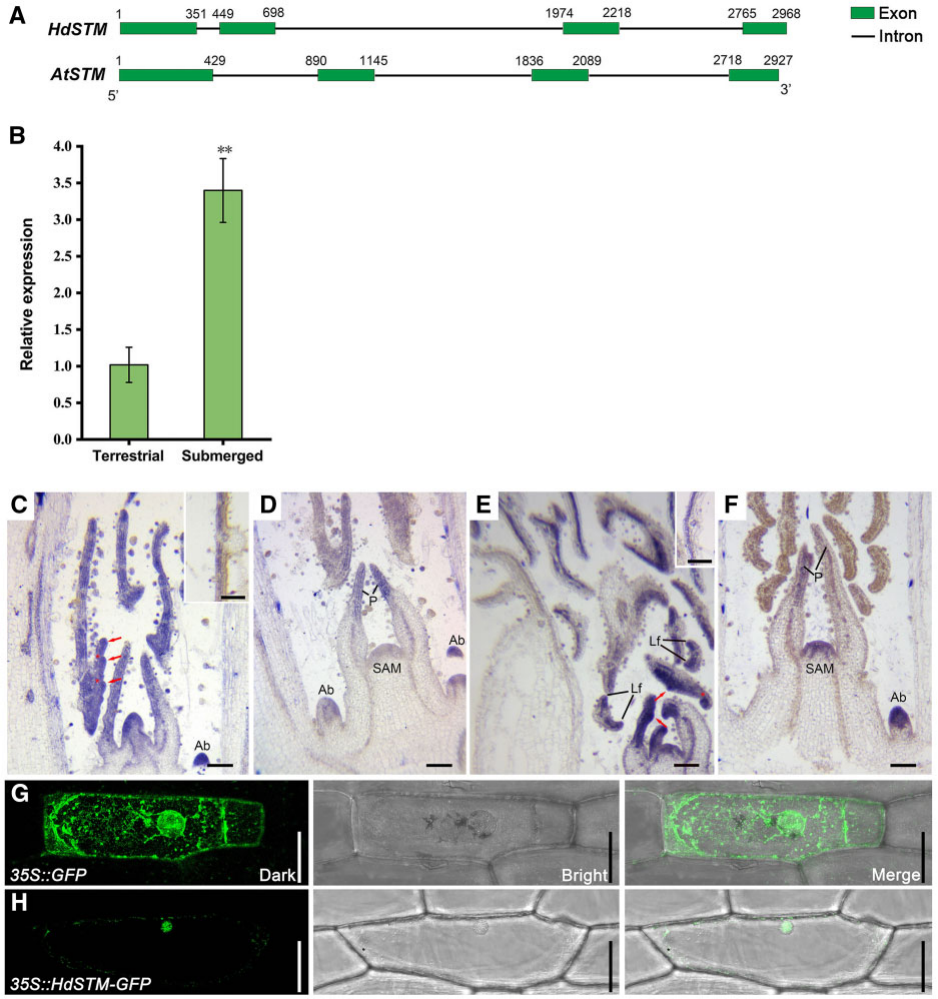
Expression analysis and subcellular localization of *HdSTM*. A, Gene structures of *HdSTM* and *AtSTM*. Both *HdSTM* and *AtSTM* contain four exons and three introns. Green boxes represent exons, and black lines represent introns. Numbers represent the positions of exon sequences. B, Expression of *HdSTM* in the shoot of *H. difformis* under terrestrial or submerged conditions. Error bars represent ± sd (*n* = 3). Asterisks indicate a significant difference relative to terrestrial shoot (Student’s *t* test: ^**^P < 0.01). C, RNA in situ hybridization of *HdSTM* in a terrestrial shoot. Note that expression of *HdSTM* can be detected in the serration (arrows) and boundary (stars) of terrestrial leaves. D, RNA in situ hybridization of *HdSTM* in SAM and leaf primordia from a terrestrial shoot apex. E, RNA in situ hybridization of *HdSTM* in a submerged shoot. Note that expression of *HdSTM* can be detected in the emerging leaflet (arrows) and boundary (star) of submerged leaves. F, RNA in situ hybridization of *HdSTM* in SAM and leaf primordia from a submerged shoot apex. Local images in (C) and (E) showed the adaxial location of *HdSTM* in developing leaves. P, primordia; Lf, leaflet primordia; Ab, axillary buds. G and H, Subcellular localization performed in onion showed that HdSTM mainly localized to the nucleus. GFP driven by the 35S promoter was used as a control. GFP is shown in green. The left, middle, and right panels show darkfield, brightfield, and merged images. Bars = 0.5 mm in (C–F). Bar = 50 μm in (G and H).

Previous studies have shown that *STM* and its orthologs are pretty important for leaf development (Nishii et al., 2017; Kierzkowski et al., 2019), and the expression pattern of *RaSTM* substantially changed in the heterophyllous plant *R. aquatica* (Nakayama et al., 2014). However, no genetic methods have been performed yet to study the role of *STM* homologs in the heterophylly of *H. difformis*. To explore the functions of the *STM* homologs in *H. difformis*, we designed degenerate primers based on the conserved regions, and the full-length cDNA of *HdSTM* was isolated using 5′ and 3′ RACE. *HdSTM* is 1,050-bp long, with four exons and three introns, which is similar to the structure of *AtSTM* in *A. thaliana* (Figure 2A).

KNOX1 contains four conserved regions that are required for its function (Gao et al., 2015). We performed amino acid sequence alignment of STM homologs from *H. difformis* (HdSTM), *A. thaliana* (AtSTM), *Nicotiana tabacum* (NtSTM), *Sesamum indicum* (SiSBH1), *Streptocarpus rexii* (SrSTM1), *Monophyllaea glabra* (MgSTM), *C. hirsuta* (ChSTM), and *Polypleurum stylosum* (PsSTM) using CLUSTALW in the MEGA 11 software package. HdSTM shares 58.88%, 58.88%, 61.41%, 64.05%, 68.11%, 69.21%, and 80.33% sequence similarity with AtSTM, ChSTM, PsSTM, NtSTM, SrSTM1, MgSTM, and SiSBH1, respectively; all of these proteins contain four conserved domains (Supplemental Figure S1). Phylogenetic analysis indicated that HdSTM clustered in the STM clade, separated from other KNOX proteins (Supplemental Figure S2).

### The expression pattern of *HdSTM* changed with a terrestrial or submerged condition

In *A. thaliana*, the activity of *STM* is mainly limited to SAM and inhibited in lateral organs (Lincoln et al., 1994; Long et al., 1996). However, this gene is expressed in the developing leaves of plant species with compound leaves (Hay and Tsiantis, 2006). To detect the expression of *HdSTM* in terrestrial or submerged conditions, we performed reverse transcription–quantitative PCR (RT–qPCR) in different organs. We found that *HdSTM* was highly expressed in the terrestrial shoot, stem, and flower and had a higher expression in submerged shoot and stem (Supplemental Figure S3). As morphological differences emerged in the early stage of the shoot (Li et al., 2017), we compared the expression of *H. difformis* in the terrestrial and submerged shoot of *H. difformis*. We found that *HdSTM* has a significantly higher expression in the submerged shoot (Figure 2B). For further analysis, we carried out RNA in situ hybridization to determine the spatiotemporal expression pattern of *HdSTM* in terrestrial and submerged shoots. *HdSTM* was expressed in the shoot meristem, leaf primordia, axillary buds, and the stem cortex beneath the meristem in terrestrial and submerged shoots (Figure 2, C–F). Notably, *HdSTM* can be detected in the serration and boundary of terrestrial leaves and in emerging leaflets (Figure 2C) and in the boundary of submerged leaflets (Figure 2E). In the developing leaves, *HdSTM* expression is confined to the adaxial region, indicating its role in adaxial development (Figure 2, C and E). Compared with terrestrial primordia, the staining in the SAM on the land plant is very faint (Figure 2D). Conversely, when compared with the staining in submerged leaf primordia, the expression in the SAM on the submerged plant appears to be increased (Figure 2F). We also performed RNA in situ hybridization with a sense probe, and no signal was detected (Supplemental Figure S4). In addition, subcellular localization analysis indicated that HdSTM mainly localized to the nucleus, with scattered signals on the cell membrane (Figure 2, G and H).

Two conserved noncoding sequences (CNSs), the K-box and the RB-box, in the promoter repress *STM* expression among species (Uchida et al., 2007; Aguilar-Martinez et al., 2015). To identify and investigate the regulation of these two CNSs on *HdSTM*, we cloned the 1.8-kb region upstream of the *HdSTM* promoter and identified the K-box and the RB-box in this region (Supplemental Table S1). As a starting point, we used the 1.8-kb sequence to drive the expression of the GUS reporter and transformed *A. thaliana* with this construct (Supplemental Figure S5). We found that deletion of the RB-box led to GUS expansion, while deletion of the K-box or both these two CNSs caused the GUS restriction. Furthermore, GUS signals in all transformed *A. thaliana* were increased under submerged treatment, indicating the independence of K-box and the RB-box on *HdSTM* in the submerged condition.

### Heterologous ectopic expression of *HdSTM* in *A. thaliana* leads to the leaf form change

**Figure 3:**
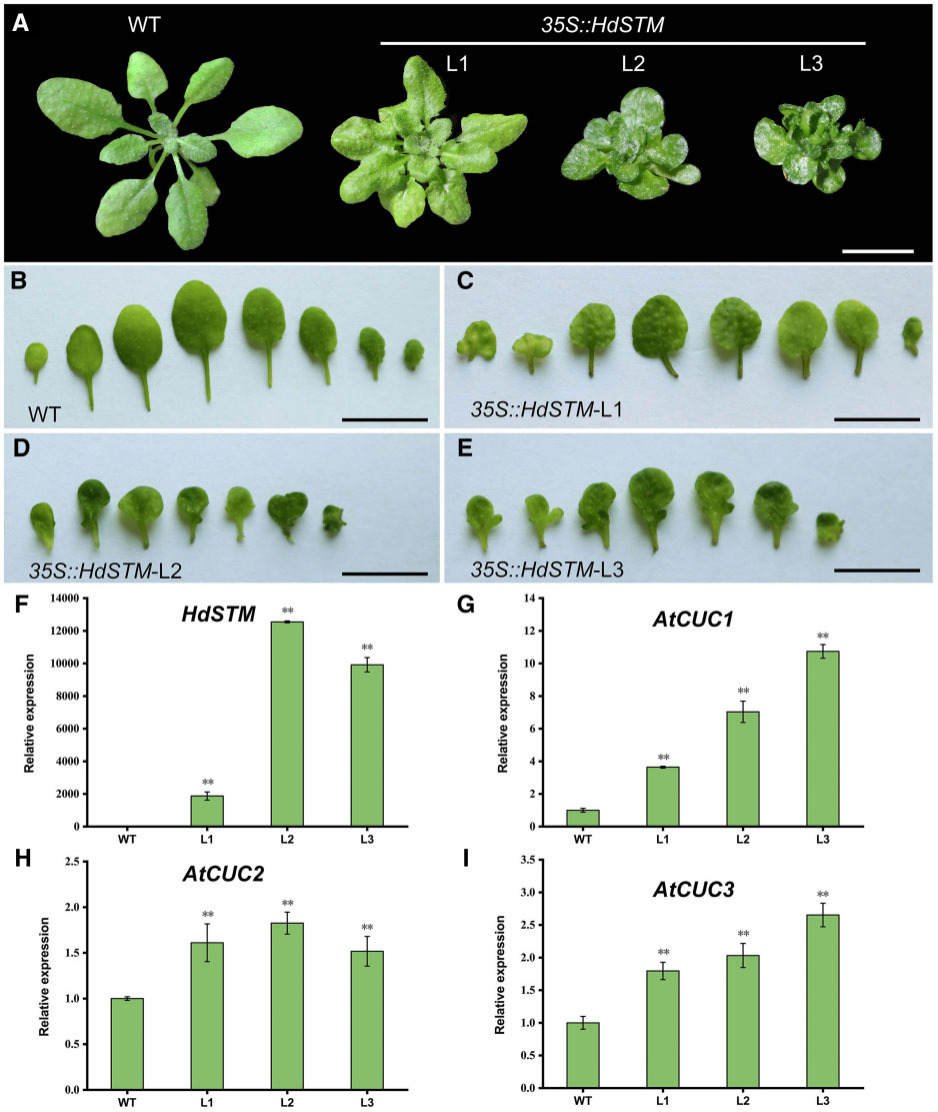
Heterologous ectopic expression of *HdSTM* in *A. thaliana*. A–E, Phenotypes of whole plants (A) and leaves (B–E) of WT and *35S::HdSTM* transgenic plants. Images in (A) were digitally extracted from original pictures for comparison. Note that *35S::HdSTM* rosette leaves developed shorter petioles and lobed leaf margins than the WT’s shallow, serrated leaves. F–I, Expression analysis of *HdSTM* and *AtCUCs* in WT and transgenic plants by RT–qPCR. Error bars represent ± sd (*n* = 3). Asterisks indicate a significant difference relative to WT (Dunnett’s test: ^**^P < 0.01). Bars = 1 cm in (A–E).

*KNOX1* genes play conserved roles in regulating leaf development, as the heterologous expression of *KNOX1* genes such as *KN1* from maize (*Zea mays*) and *CrKNOX1* from *Ceratopteris richardii* increased leaf complexity in *A. thaliana* (Sano et al., 2005). However, the functions of *STM* and its orthologs are different between diverse species. For example, the activity of *STM* was restricted in SAM and does not have a solid autonomous function in promoting leaf dissections in both *A. thaliana* and citrus (Zeng et al., 2022), while overexpressed *POTH15* (*STM* ortholog in potato (*Solanum tuberosum*)) in potato developed curved mouse-ear-shaped leaflets. Their petiole and rachis were also severely shortened (Mahajan et al., 2016). To study the role of *HdSTM* in leaf development, we first introduced the *35S::HdSTM* construct into *A. thaliana* and obtained 13 *35S::HdSTM* transgenic lines. The rosette leaves of the transformed plants developed lobed leaf margins, representing distinct phenotypic changes compared to the shallow, serrated leaves of the WT (Figure 3, A–E). We also found shorter petioles in transgenic lines than in the WT, similar to the described phenotype in potato (Mahajan et al., 2016). It was reported that *CUCs* are involved in determining leaf complexity, and *STM* activates *CUC* expression (Aida et al., 1999; Spinelli et al., 2011). Therefore, we then detected *CUC* expression in the *35S::HdSTM* transgenic lines. As expected, the expression of *AtCUC1*, *AtCUC2*, and *AtCUC3* was significantly induced in the transgenic plants compared to the WT (Figure 3, F–I).

### Overexpression of *HdSTM* induced quick leaf variations of *H. difformis* in submergence

**Figure 4:**
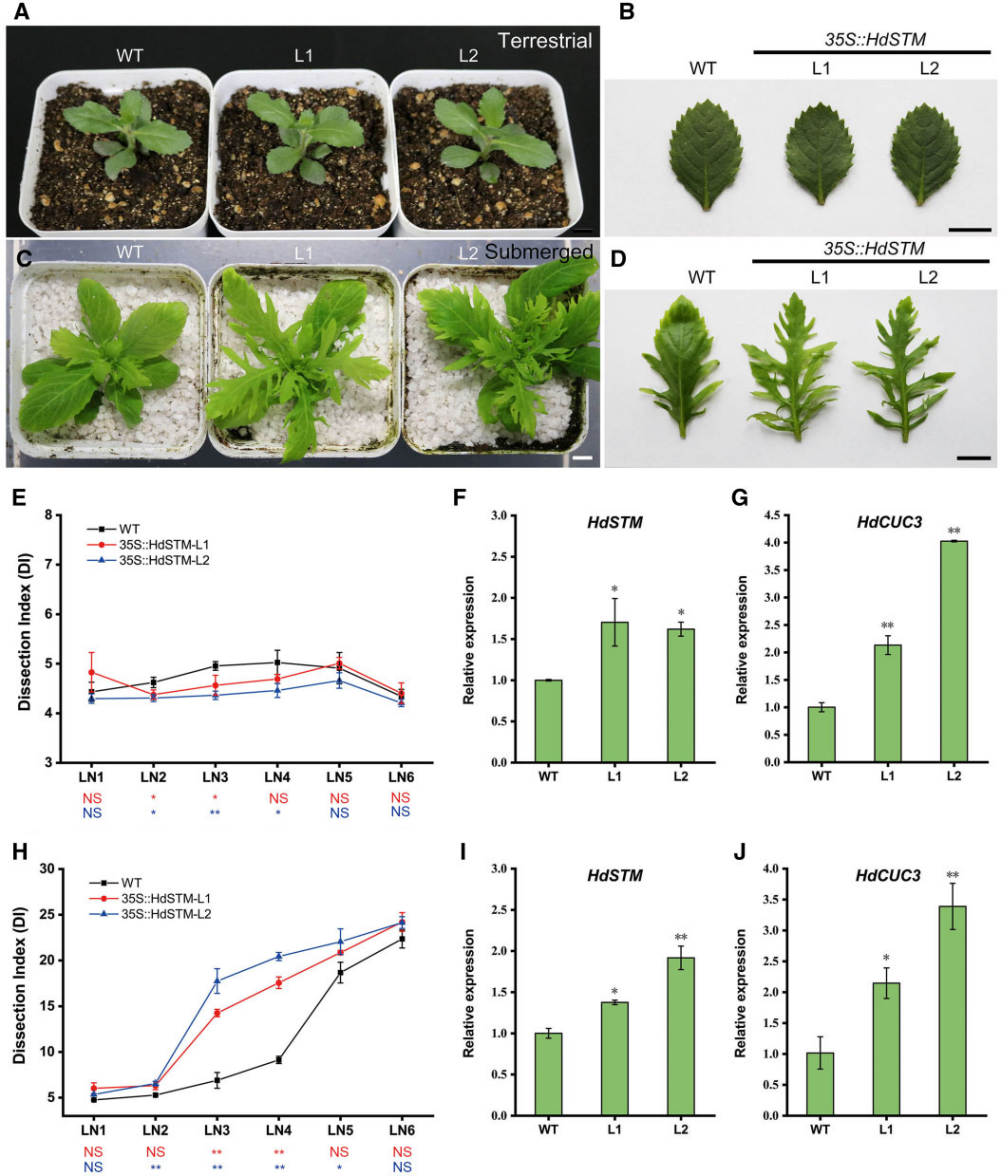
Overexpression of *HdSTM* in *H. difformis*. Phenotypes of whole plants (A) and leaves (B) of WT and *35S::HdSTM* transgenic plants in terrestrial conditions. C, Phenotypes of whole plants and LN3 stage leaves (D) of WT and *35S::HdSTM* transgenic plants under submerged treatment for 2 weeks. Note that emerging leaves of transgenic lines are more complex than WT. E, DI of WT and *35S::HdSTM* transgenic plants in terrestrial conditions. Error bars represent ± sd (*n* = 3). Leaf number (LN) was assigned to each emerged leaf after the start of the submergence experiment. The symbols below the *X*-axis indicate statistical differences between transgenic plants and WT. (Up, L1 and WT; Down, L2 and WT; Dunnett’s test: ^*^P < 0.05; ^**^P < 0.01; NS, not significant). F, Expression analysis of *HdSTM* in WT and *35S::HdSTM* transgenic plants grow in terrestrial conditions by RT–qPCR. Error bars represent ± sd (*n* = 3). Asterisks indicate a significant difference relative to terrestrial shoot of WT (Dunnett’s test: ^*^P < 0.05). G, Expression analysis of *HdCUC3* in WT and *35S::HdSTM* transgenic plants grow in terrestrial condition by RT–qPCR. Error bars represent ± sd (*n* = 3). Asterisks indicate a significant difference relative to terrestrial shoot of WT (Dunnett’s test: ^**^P < 0.01). H, DI of WT and *35S::HdSTM* transgenic plants in submerged condition. Error bars represent ± sd (*n* = 3). The symbols below the *X*-axis indicate statistical differences between transgenic plants and WT. (Up, L1 and WT; Down, L2 and WT; Dunnett’s test: ^*^P < 0.05; ^**^P < 0.01; NS, not significant). I, Expression analysis of *HdSTM* in WT and *35S::HdSTM* transgenic plants grow in submerged conditions by RT–qPCR. Error bars represent ± sd (*n* = 3). Asterisks indicate a significant difference relative to submerged shoot of WT (Dunnett’s test: ^*^P < 0.05; ^**^P < 0.01). J, Expression analysis of *HdCUC3* in WT and *35S::HdSTM* transgenic plants grow in submerged conditions by RT–qPCR. Error bars represent ± sd (*n* = 3). Asterisks indicate a significant difference relative to submerged shoot of WT (Dunnett’s test: ^*^P < 0.05; ^**^P < 0.01). Bars = 1 cm in (A–D).

We then transformed *H. difformis* with the *35S::HdSTM* construct and obtained 10 *35S::HdSTM* transgenic lines. However, both WT and transgenic lines formed broad, serrated leaf margins in terrestrial condition (Figure 4, A and B), and their dissection index (DI) is almost at the same level (Figure 4E). To detect the expression of *HdSTM* in WT and transgenic plants, we performed RT–qPCR in terrestrial shoots and found that transgenic plants have a higher expression than WT (Figure 4F). In heterophyllous plant *R. aquatica*, the expression of *STM* and *CUC3* changed with ambient surroundings. Both of them were upregulated in complex leaves (Nakayama et al., 2014), indicating an important role of *CUC3* in heterophylly. Therefore, we measured *HdCUC3* expression in the WT and transgenic plants and found that *HdCUC3* was also significantly upregulated in the transgenic plants (Figure 4G). To verify our hypothesis that *HdSTM* may have a different role in leaf form under submerged conditions, we shifted WT and *35S::HdSTM* transgenic plants to submerged conditions for one month. We found that all plants finally developed deep lobed leaves, and their DI is almost at the same level after 1-month of treatment (Figure 4H). Interestingly, the *35S::HdSTM* lines have quick leaf variations in submergence. DIs of transgenic plants are significantly increased at the LN3 and LN4 stages (2 weeks after submergence) than the WT (Figure 4, C and D), suggesting that *HdSTM* plays a role in the complex leaf formation in submerged conditions. RT–qPCR showed that *HdSTM* and *HdCUC3* expression were also significantly upregulated in transgenic lines under submerged conditions (Figure 4, I and J).

### Leaf development and heterophylly are disturbed in *HdSTM*-RNAi transgenic *H. difformis*

**Figure 5:**
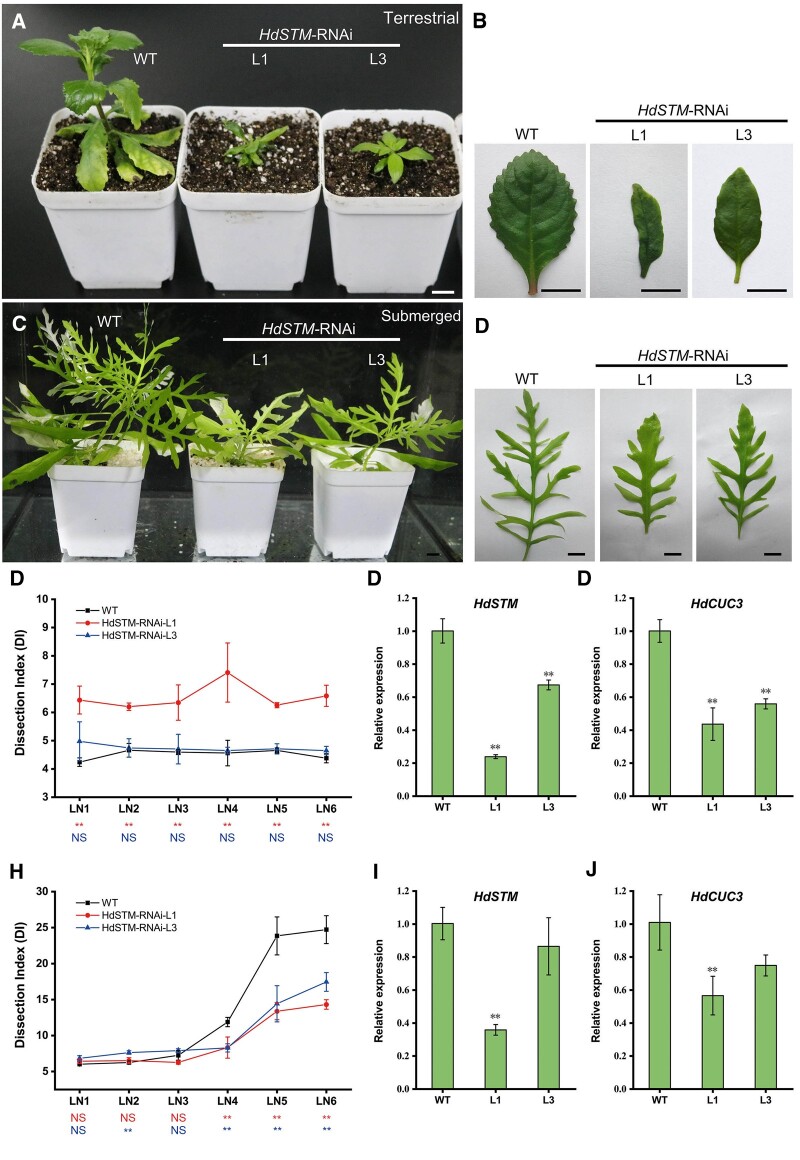
Knockdown of *HdSTM* in *H. difformis*. Phenotypes of whole plants (A) and leaves (B) of WT and *HdSTM*-RNAi transgenic plants in terrestrial conditions. Note that phyllotaxy and leaf shape are significantly altered in transgenic plants. C, Phenotypes of whole plants and leaves (D) of WT and *HdSTM*-RNAi transgenic plants under submerged conditions for 1 month. Note that WT leaves are more complex than transgenic leaves. E, DI of WT and *HdSTM*-RNAi transgenic plants in terrestrial conditions. Error bars represent ± sd (*n* = 3). LN was assigned to each emerged leaf after the start of the submergence experiment. The symbols below the *X*-axis indicate statistical differences between transgenic plants and WT. (Up, L1 and WT; Down, L3 and WT; Dunnett’s test: ^*^P < 0.05; ^**^P < 0.01; NS, not significant). F, Expression analysis of *HdSTM* in WT and *HdSTM*-RNAi transgenic plants grow in terrestrial conditions by RT–qPCR. Error bars represent ± sd (*n* = 3). Asterisks indicate a significant difference relative to terrestrial shoot of WT (Dunnett’s test: ^**^P < 0.01). G, Expression analysis of *HdCUC3* in WT and *HdSTM*-RNAi transgenic plants grow in terrestrial conditions by RT–qPCR. Error bars represent ± sd (*n* = 3). Asterisks indicate a significant difference relative to terrestrial shoot of WT (Dunnett’s test: ^**^P < 0.01). H, DI of WT and *HdSTM*-RNAi transgenic plants in submerged conditions. Error bars represent ± sd (*n* = 3). The symbols below the *X*-axis indicate statistical differences between transgenic plants and WT. (Up, L1 and WT; Down, L3 and WT; Dunnett’s test: ^*^P < 0.05; ^**^P < 0.01; NS, not significant). I, Expression analysis of *HdSTM* in WT and *HdSTM*-RNAi transgenic plants grow in submerged conditions by RT–qPCR. Error bars represent ± sd (*n* = 3). Asterisks indicate a significant difference relative to submerged shoot of WT (Dunnett’s test: ^**^P < 0.01). J, Expression analysis of *HdCUC3* in WT and *HdSTM*-RNAi transgenic plants grow in submerged conditions by RT–qPCR. Error bars represent ± sd (*n* = 3). Asterisks indicate a significant difference relative to submerged shoot of WT (Dunnett’s test: ^**^P < 0.01). Bars = 1 cm in (A–D).

To further investigate the function of *HdSTM* in *H. difformis*, we designed an RNAi construct (*HdSTM*-RNAi) containing the 5- to 275-bp coding sequence of *HdSTM* and used it to transform *H. difformis*. We obtained 15 transgenic lines and recorded the phenotypes of two as their representative (Figure 5). In terrestrial condition, WT plants displayed broad, serrated leaves, whereas line 1 (L1, the strong phenotype) of the transgenic plants exhibited shallow-waved margins, a sickle-like leaf form, and disturbed phyllotaxy. Transgenic line 3 (L3, the moderate phenotype) also showed a shallow-waved leaf margin in terrestrial condition (Figure 5, A and B). Quantitative results of leaf complexity showed that transgenic L1 has a higher DI value, while WT and L3 are almost at the same levels (Figure 5E). We then measured *HdSTM* and *HdCUC3* expression in the terrestrial shoot of WT and transgenic plants and found that they were significantly downregulated in transgenic lines (Figure 5, F and G). Subsequently, we shifted WT and *HdSTM*-RNAi transgenic plants to submerged conditions for one month. Although all plants still developed lobed submerged leaves, the *HdSTM*-RNAi lines showed simplified leaf form (Figure 5, C and D) and reduced leaf complexity at the LN4 to LN6 stage than the WT (Figure 5H). We found that *HdSTM* and *HdCUC3* expression were also significantly downregulated in transgenic L1 under submerged conditions (Figure 5, I and J). In addition, the expression of *HdSTM* is significantly lower in transgenic L1 than L3 in both terrestrial and submerged conditions (Dunnett’s test, *P* < 0.01), which may be relevant to its severe phenotype than L3.

### HdSTM physically interacts with HdCUC3

**Figure 6:**
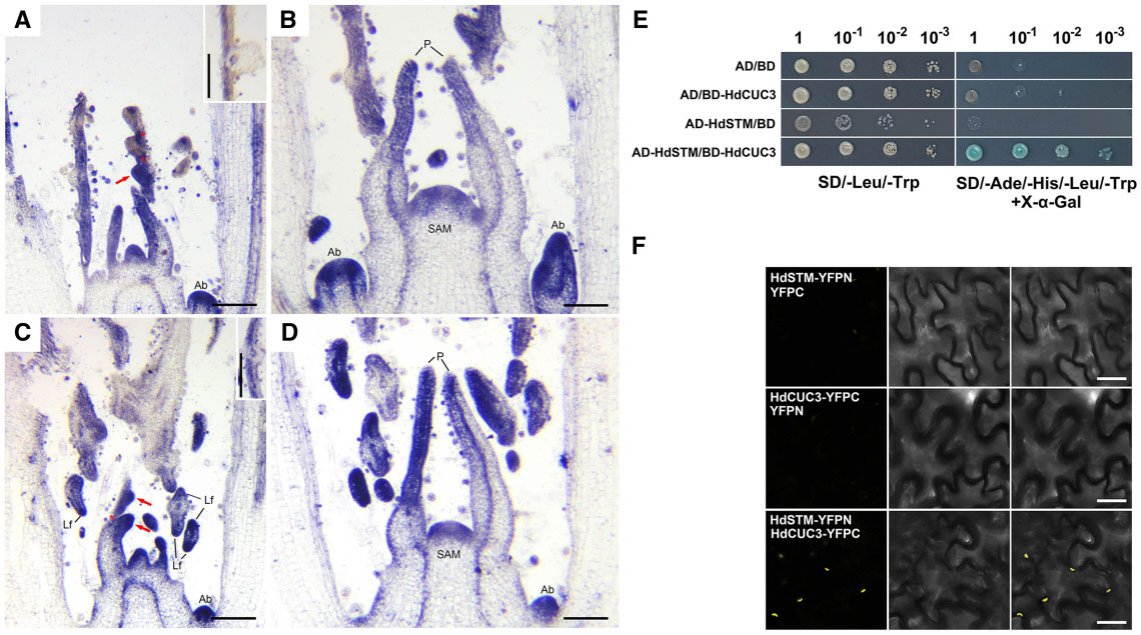
Expression pattern of *HdCUC3* and physical interactions between HdSTM and HdCUC3. A, RNA in situ hybridization of *HdCUC3* in a terrestrial shoot. Note that expression of *HdCUC3* can be detected in the serration (arrow) and boundary (stars) of terrestrial leaves. B, RNA in situ hybridization of *HdCUC3* in SAM and leaf primordia from a terrestrial shoot apex. C, RNA in situ hybridization of *HdCUC3* in a submerged shoot. Note that expression of *HdCUC3* can be detected in the emerging leaflet (arrows) and boundary (star) of submerged leaves. D, RNA in situ hybridization of *HdCUC3* in SAM and leaf primordia from a submerged shoot apex. Local images in (A) and (C) showed the adaxial location of *HdCUC3* in developing leaves. P, primordia; Lf, leaflet primordia; Ab, axillary buds. E, Yeast two-hybrid assay showing that HdSTM interacts with HdCUC3. F, BiFC confirms that HdSTM interacts with HdCUC3 in the nucleus. Darkfield, brightfield, and merged channels are shown successively from left to right. Bars = 0.5 mm in (A–D). Bars = 25 μm in (F).

*STM* activates *CUC* expression (Spinelli et al., 2011; Balkunde et al., 2017) and the expression of *RaCUC3* also changes with ambient surroundings in *R. aquatica* (Nakayama et al., 2014). To detect the expression of *HdCUC3* in *H. difformis*, we cloned the cDNA of *HdCUC3* (Supplemental Figures S6 and S7) and performed RNA in situ hybridization in terrestrial and submerged shoots. Surprisingly, unlike the restricted expressions of *CUC3* at the boundary between shoot apex and leaf primordia (Blein et al., 2008) and in the boundary domain between the leaflet primordia (Nakayama et al., 2014), *HdCUC3* was broadly expressed in the shoot meristem, leaf primordia, axillary buds, stem cortex beneath the meristem in both terrestrial and submerged shoots (Figure 6, A–D). We also found that the expression pattern of *HdCUC3* in terrestrial primordia and SAM is similar to the staining in submerged leaf primordia and SAM (Figure 6, B and D). In addition, *HdCUC3* was detected in the serration and boundary of terrestrial leaves and submerged leaflets (Figure 6, A and C), which is similar to the spatiotemporal expression pattern with *HdSTM* (Figure 2).

KNOX1 physically interacts with other proteins during leaf development in tomato (*Solanum lycopersicum*) (Kimura et al., 2008). CUC proteins can also physically interact with each other, and the resulting complexes promote leaf complexity (Rubio-Somoza et al., 2014; Goncalves et al., 2015). However, to our knowledge, no previous studies have detected the direct interaction of KNOX1 and CUC proteins. As we found that *HdSTM* and *HdCUC3* have the same spatiotemporal expression pattern and *HdCUC3* changed with *HdSTM* in transgenic plants, we performed two-hybrid competition assays of HdSTM and HdCUC3 to see whether they have physical interaction. We found that HdSTM directly interacted with HdCUC3, while HdCUC3 or HdSTM did not activate the reporter genes in yeast (Figure 6E). To verify that HdSTM and HdCUC3 interact in vivo, we performed a bimolecular fluorescence complementation assay (BiFC) assay using the abaxial leaf epidermis of *Nicotiana benthamiana*. HdCUC3-YFPC - YFPN and HdSTM-YFPN-YFPC were used as negative controls (Girin et al., 2011). The fluorescent signals of yellow fluorescent protein (YFP) were observed upon co-infiltration of HdCUC3 and HdSTM (Figure 6F), indicating that HdSTM directly interacts with HdCUC3 at the protein level.

### The expression of *HdSTM* is upregulated by ethylene

**Figure 7:**
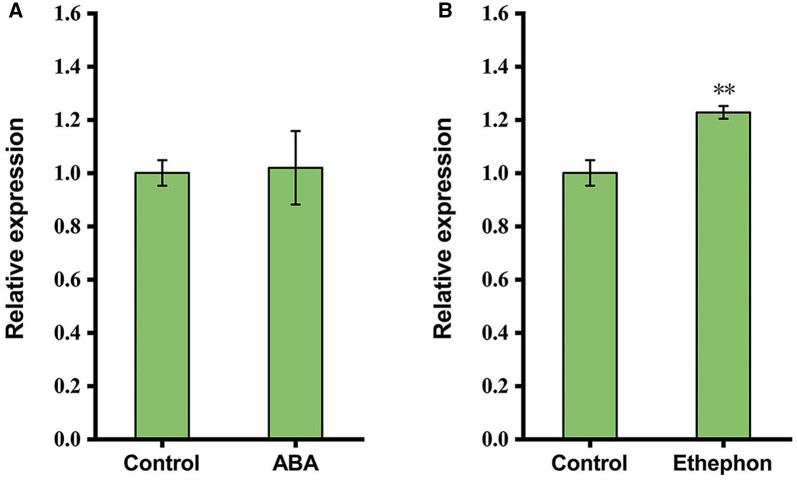
Expression of *HdSTM* under phytohormone treatments. A, Expression of *HdSTM* under 100 µM ABA treatment. B, Expression of *HdSTM* under 100-µM ethephon treatment. Error bars represent ± sd (*n* = 3). Asterisks indicate significant difference relative to control (Student’s *t* test: ^**^P < 0.01).

Phytohormones are stimulated by different environmental conditions and the theory that phytohormones achieve the morphogenesis induced by the environment has been summarized by many reviewers (Kuwabara et al., 2003; Su et al., 2011; Vanstraelen and Benkova, 2012; Nakayama et al., 2017). Among those phytohormones, ethylene and ABA are well-known to function antagonistically in heterophylly, as ethylene promotes submerged leaf formation while ABA induced terrestrial leaves (Wanke, 2011; Kim et al., 2018; Koga et al., 2021). Our previous study also verified that exogenous ethylene promotes the dissected leaf formation, while ABA induces simplified leaf form in *H. difformis* (Li et al., 2017). To see the effects of ABA and ethylene on the expression of *HdSTM*, we performed exogenous ABA and ethylene treatment and detected the expression by RT–qPCR. We found that the expression of *HdSTM* did not change under ABA treatment but was significantly induced by ethylene (Figure 7), indicating that *HdSTM* might be involved in the ethylene mediated heterophylly in *H. difformis*.

## Discussion

The expression pattern of a gene is closely associated with its function, and gene expression is usually regulated by upstream CNSs (Inada et al., 2003). In *A. thaliana*, *KNOX1* expression is restricted to the SAM throughout leaf development, resulting in the formation of simple leaves. In *C. hirsuta*, *KNOX1* genes are activated in leaf primordia, leading to complex leaf formation (Hay and Tsiantis, 2006; Canales et al., 2010). Recently, the time-lapse imaging analysis of *C. hirsuta* has revealed the function of *ChSTM* influences leaf form by slowing growth and delaying differentiation in the proximal domain where serration occurs (Kierzkowski et al., 2019). Here, we verified that *HdSTM* was expressed in many organs of *H. difformis* (Supplemental Figure S3) and was significantly upregulated in shoots grown in submerged conditions (Figure 2B). RNA in situ hybridization showed that the expression of *HdSTM* was broadly detected in shoots, which is similar to the expression pattern of *STM* orthologs in Bignonieae species (Sousa-Baena et al., 2014). It is worth mentioning that these *STM* orthologs were detected in the adaxial domain of lateral branches of tendrils (Sousa-Baena et al., 2014), similar to the adaxial distribution of *HdSTM* in developing leaves (Figure 2), suggesting their potential roles in the adaxial development of tendrils and leaves between species.

The different leaf dissection patterns between the sister species *Capsella rubella* and *Capsella grandiflora* are due to variations in the cis-regulatory regions of the homeobox gene *REDUCED COMPLEXITY*, which alter its activity in the developing lobes of the leaf (Sicard et al., 2014). It was reported that RB-boxes and K-boxes are conserved binding motifs in *STM* promoters. Deletion of the RB-box in *STM* led to its expanded expression in the *A. thaliana* hypocotyl and root (Aguilar-Martinez et al., 2015). In addition, deletion of the K-box in the *NTH15* gene (tobacco (*N. tabacum*) ortholog of *A. thaliana STM*) resulted in its expanded expression in the basal regions of tobacco leaves (Uchida et al., 2007). Here, we determined that the roles of the RB-box and K-box of *HdSTM* are likely antagonistic, as deletion of the RB-box led to the expansion of *HdSTM* expression, while deletion of the K-box limited *HdSTM* expression (Supplemental Figure S5). However, GUS signals in all transformed *A. thaliana* were increased under submerged treatment, indicating the independence of K-box and the RB-box on *HdSTM* in the submerged condition.

The overexpression of *KNOX1* genes in tomato, lettuce (*Lactuca sativa*), and strawberry (*Fragaria ananassa*) resulted in ultracompound or deeply serrated leaves (Hareven et al., 1996; Frugis et al., 2001; Kimura et al., 2008; Chatterjee et al., 2011). We ectopically expressed *HdSTM* in *A. thaliana* and found that these *35S::HdSTM* plants showed significantly increased leaf complexity and *CUC* transcript levels (Figure 4), pointing to conserved roles for *HdSTM* in leaf development and gene regulation. However, *H. difformis* plants overexpressing *HdSTM* showed no obvious phenotypic changes in terrestrial conditions (Figure 4). These results indicated that *HdSTM* is functionally conserved but works differently depending on plants. In addition, we found the upregulation in the overexpressed *H. difformis* is no more than five-fold, which may be due to the limited function of the 35S promoter in different species (Chen et al., 2021), and overexpression by the different promoters could result in significant differences in expression levels and phenotypes (Shani et al., 2009). Correspondingly, knockdown of *HdSTM* led to disturbed leaf development in terrestrial conditions and weakened heterophylly in submerged conditions (Figure 5). Since the aspect ratio affects the calculation of leaf complexity (Rupp and Gruber, 2019), the DIs in these terrestrial leaves seems inconsistent with the images and may not be suitable for quantifying these narrow leaves. For future studies, researchers need to optimize the quantification of leaf complexity via multiple methods (Wu et al., 2016; Rupp and Gruber, 2019). Previous studies have reported a lot of phenotypes of the overexpression or knockdown of *KNOXI* genes in diverse species. Here we provide insights into how *HdSTM* affects the leaf development and is also involved in the submerged response in *H. difformis*. Our results indicated that *HdSTM* might not be the critical regulator but is also involved in the heterophylly of *H. difformis*. The fact that leaf deformation occurred in terrestrial conditions and serration is still occurring in submerged conditions suggested that *HdSTM* may affect leaf development of *H. difformis* in both terrestrial and submerged conditions. As KNOXI proteins participate in leaf development and work redundantly (Zeng et al., 2022), the knockdown of *HdSTM* cannot lead to the loss of heterophylly and may be due to the existence of its functionally redundant homologs.

It is known that the *CUC2*-auxin module is required for leaf serration (Nikovics et al., 2006; Rodriguez et al., 2014). However, the *A. thaliana* mutant *cuc2-1* exhibits a smooth margin as it fails to initiate leaf teeth, while the *cuc3-105* mutant also initiates serrations, but they are rapidly smoothed during leaf development, suggesting their different functions on leaf serration formation (Hasson et al., 2011; Serra and Perrot-Rechenmann, 2020). Both *STM* and *CUC3* can be induced by abiotic stress (Fal et al., 2016). However, their distributions are pretty different between diverse species. In *A. thaliana*, *CUC3* expression can be detected in the boundary between the SAM and the cotyledons (Vroemen et al., 2003) and the boundaries between leaf primordia and the shoot meristem (Hibara et al., 2006). Correspondingly, the expression of *STM* was restricted to the SAM and absent from leaf primordia (Long et al., 1996). In the compound-leaf plant *C. hirsuta*, orthologs of *STM* were observed in the outer cell layers of leaf primordia, the SAM and the abaxial side of young leaves (Hay and Tsiantis, 2006), while orthologs of *CUC3* were expressed at the boundary of leaflet primordia during leaflet initiation (Blein et al., 2008). In *R. aquatica*, *RaSTM* was upregulated at the base of developing leaf primordia in plants that formed complex leaf shapes. *RaCUC3* was also upregulated in the boundary domain between the leaflet primordia (Nakayama et al., 2014). Here, we found that *HdSTM* and *HdCUC3* have an overlapped spatiotemporal expression pattern in *H. difformis* (Figures 2 and 6), and the expression of *HdCUC3* is closely associated with the expression changes of *HdSTM* in transgenic plants of *H. difformis*. These results indicated that *HdCUC3* has a close relationship with *HdSTM* in *H. difformis*. Because *CUCs* play conserved roles in the leaflet or serration formation (Li et al., 2021), the *HdSTM* involved in leaf development or heterophylly may go through the regulation of *CUCs*, probably via *HdCUC3*. However, due to the expression pattern of *HdCUC3* being different from that of previously reported plants with compound leaves (Blein et al., 2008), the hypothesis needs to be carefully considered in the future. To verify the expression level of *HdCUC3*, we also performed RT–qPCR to detect the expression levels of *HdCUC3* in terrestrial and submerged conditions. However, they showed no significant difference between these two conditions (Student’s t test, *P* < 0.05) (Supplemental Figure S8). These results indicated that *HdCUC3* might not be sensitive to terrestrial/submerged conditions, or regulators may complexly regulate the expression of *HdCUC3* in different conditions.

KNOX1 transcription factors form complexes with BELL1-like proteins to regulate leaf development (Hake et al., 2004; Hay and Tsiantis, 2010); CUC proteins also physically interact with each other, and the resulting complexes promote leaf complexity (Rubio-Somoza et al., 2014; Goncalves et al., 2015). Here, we detected a physical interaction between HdSTM and HdCUC3 both in vitro and in vivo (Figure 6), pointing to a potential function of the HdSTM–HdCUC3 complex in *H. difformis*. For example, the constitutively photomorphogenic 1(COP1)—suppressor of phyA-105 complex functions as the negative regulator to repress the photomorphogenesis in the dark condition, while the complex was degraded and dissociated in the light condition and eventually induced downstream genes for photomorphogenic development (Hoang et al., 2019). Considering the function of STM and CUC orthologs as transcription factors, the HdSTM–HdCUC3 complex in *H. difformis* may regulate the dissociative HdSTM and HdCUC3 for their downstream genes. Future studies should determine whether this interaction is also found in other species or whether it is specific to *H. difformis*.

In numerous plant species, ethylene accumulation in plants during submergence promotes the formation of submerged leaf form in heterophyllous plants (Nakayama et al., 2017; Li et al., 2021). Our previous study showed that treating terrestrial *H difformis* plants with ethylene resulted in the formation of dissected leaves, which is similar to the submerged phenotype. Instead, applying ethylene-response inhibitor AgNO_3_ reduced the leaf complexity in different conditions (Li et al., 2017). Here we found that the expression of *HdSTM* is upregulated by ethylene, suggesting that *HdSTM* might be involved in the ethylene-mediated heterophylly in *H. difformis*.

In conclusion, we explored the roles of *HdSTM* in *H. difformis* using molecular, morphogenetic, and biochemical tools. We demonstrated that *HdSTM* is involved in the heterophylly of *H. difformis* and is regulated by phytohormones. These findings provide insights into the molecular mechanism underlying heterophylly and plant acclimation to the environment.

## Materials and methods

### Plant materials and growth conditions

*Hygrophila difformis* and *A. thaliana* were grown in growth chambers under a 16-h-light/8-h-dark cycle at 23°C with a white light flux density of 60 µmol m^−2^ s^−1^. *Arabidopsis thaliana* seeds of the Columbia ecotype were obtained from the Arabidopsis Biological Resource Center. Terrestrial *H. difformis* plants were watered every 7 days and maintained at a relative humidity of 30%. Submerged plants were grown in aquariums (35 × 20 × 35 cm) and placed in growth chambers with the temperature set at 23°C and a white light flux density of 60 µmol m^−2^ s^−1^.

### Gene cloning and gene structure analysis

Total RNA was extracted from *H. difformis* shoots using TRIzol reagent (Invitrogen, Carlsbad, CA, USA), and cDNA was synthesized from 1-µg total RNA using a Primescript RT Reagent kit (Takara, Kyoto, Japan). To isolate *HdSTM* and *HdCUC3* from *H. difformis*, degenerate primers were designed based on conserved gene regions. Full-length *HdSTM* and *HdCUC3* cDNAs were generated from total RNA via the 5′- and 3′-RACE (rapid amplification of cDNA ends) method using a RACE kit (Clontech, Mountain View, CA, USA). Genomic fragments of *HdSTM* and *HdCUC3* were cloned via PCR amplification, and the coding sequence data were deposited in the GenBank Nucleotide Sequence Databases. Gene information on *AtSTM* was obtained from The Arabidopsis Information Resource (https://www.arabidopsis.org/). Gene structure visualization of *AtSTM* and *HdSTM* was performed using GSDS version 2.0 online software (http://gsds.cbi.pku.edu.cn/). Primer information is given in Supplemental Table S2.

### Amino acid sequence alignment and phylogenetic analysis

The full-length amino acid sequences of STM and CUC3 from various species were obtained via NCBI (https://www.ncbi.nlm.nih.gov/). Amino acid sequence alignment was performed using ClustalW software (Thompson et al., 1994). Phylogenetic analysis was performed via the neighbor-joining method with 1,000 bootstrap replicates using MEGA version 11 software (Saitou and Nei, 1987). Information about the gene sequences used for amino acid sequence alignment and phylogenetic analysis is given in Supplemental Table S3.

### RT–qPCR

Total RNA was extracted from shoots, including leaf primordia of plants grown for a month and used to synthesize cDNA, as described previously (Nakayama et al., 2014). Shoot tips, including SAM and primordia up to stage P4 were collected. The leaf morphology at stage P3 is starting to be distinguished under different environmental conditions (Li et al., 2017), and P4 stage is easy to be distinguished. For gene expression in different organs, their relative expression was normalized to terrestrial or submerged mature leaves (at stage P6). RT–qPCR was performed using the SYBR Premix Ex Taq Kit (Takara, Kyoto, Japan) in a CFX96 Real Time PCR system (Bio-Rad, Berkeley, CA, USA). Experiments were performed in triplicate from three independent tissue RNA extractions. *HdACTIN1* (*HdACT1*) and *AtACTIN2* (*AtACT2*) were used as internal references for *H. difformis* and *A. thaliana*. The 2^−ΔΔCt^ method was employed to calculate the genes’ relative expression (Pfaffl, 2001). Gene expression was normalized to the WT or control. Primer information is given in Supplemental Table S2.

### In situ hybridization

Shoots of *H. difformis* grown in terrestrial or submerged conditions were fixed in formol-acetic-alcohol, and in situ hybridization was performed as previously described (Nakayama et al., 2012). Primers targeted for the unique region of HdSTM (530–651 bp) and HdCUC3 (269–393 bp) were used for PCR amplification to synthesize the sense and antisense probes using SP6 and T7 polymerase, respectively. Primer information is given in Supplemental Table S2.

### Subcellular localization

The green fluorescent protein (*GFP*) sequence in pCAMBIA1302 was fused with the *HdSTM* coding sequence without the stop codon via the NcoI and SpeI cleavage sites using the Trelief SoSoo Cloning Kit (Tsingke Biotechnology Co, Beijing, China). Subcellular localization was performed in onion (*Allium cepa*) epidermal cells as previously described (Zhao et al., 2018). Primer information is given in Supplemental Table S2.

### Heterologous expression of *HdSTM* in *A. thaliana*

The full-length coding sequence of *HdSTM* was amplified and cloned into the pMYC vector (Heenatigala et al., 2020) via the PstI and SalI cleavage sites to generate the gene overexpression construct (*35S::HdSTM*). The construct was transferred into *A. tumefaciens* GV3101 by electroporation and transformed into *A. thaliana* via the floral dip method (Clough and Bent, 1998). Transgenic plants were selected on Murashige and Skoog medium containing 25 mg L^−1^ hygromycin B. Primer information is given in Supplemental Table S2.

### Analysis of the *HdSTM* promoter in *A. thaliana*

The 5′-upstream promoter region of *HdSTM* (−1799 to 0 bp) was obtained by genome walking using a Genome Walking kit (Takara) and cloned into pCAMBIA1301 via the XbaI and NcoI cleavage sites to generate the *pHdSTM::GUS* plasmid. The plasmid was transferred into *Agrobacterium* GV3101 and transformed into *A. thaliana* for further analysis. To identify the functions of two CNSs (RB-box and K-box motif) in the *HdSTM* promoter, constructs with internal deletions (RB-box [−848 to −769 bp], K-box [−387 to −289 bp], or both) in the *HdSTM* promoter were generated using overlapping PCR. For GUS staining of transformed *A. thaliana*, 10-day-old seedlings were examined immediately. For submersion treatment of *A. thaliana*, 10-day-old seedlings were submerged in the aquarium for 24 h and analyzed by GUS staining (Li et al., 2017). Primer information is given in Supplemental Table S2.

### Constructions and transformation of *HdSTM* in *H. difformis*

To generate the *HdSTM*-RNAi construct, the 271-bp sense and antisense fragments from the 5′-end of *HdSTM* cDNA (5–275 bp) were amplified using gene-specific primers containing XhoI (5′-end)/KpnI (3′-end) and XbaI (5′-end)/HindIII (3′-end) sites. The two fragments were separately inserted into the pHANNIBAL vector (Wesley et al., 2003). The entire RNAi cassette was subcloned into the pCAMBIA1300 binary vector through the SacI and PstI cleavage sites to construct the *HdSTM*-RNAi plasmid. The recombinant *HdSTM*-RNAi construct was transferred into *Agrobacterium* strain LBA4404 and transformed into *H. difformis* (Li et al., 2020). The full-length coding sequence of *HdSTM* was amplified and cloned into the pMYC vector (Heenatigala et al., 2020) through the PstI and SalI cleavage sites to generate the overexpression construct (*35S::HdSTM*). The *35S::HdSTM* constructs were transformed into *H. difformis* (Li et al., 2020). Primer information is given in Supplemental Table S2.

For morphological analysis, leaves were photographed with a Canon EOS80D camera, and all light microscopy observations were performed under a Sunny EX20 light microscope and photographed with a ToupCam TP605100A digital camera. The images were integrated using MvImage media software (ToupCam). Leaf complexity was estimated based on the DI, calculated as previously described (Li et al., 2017). All calculations were performed using ImageJ 1.47v (http://rsb.info.nih.gov/ij/). Statistical differences were determined using Student’s *t* test.

### Phytohormone treatments

For phytohormone treatments, a 50-µL drop of each hormone solution was applied to the shoot apex of a terrestrial plant once daily for 2 weeks. The plants were treated with 100-µM ABA or 100-µM ethephon, all in 0.1% (w/v) ethanol; the control solution comprised 0.1% (w/v) ethanol alone. Each treatment had three replicates. After 2 weeks of treatment, the shoots were harvested from all plants for gene expression analysis.

### Yeast two-hybrid assay

Full-length coding sequences of *HdSTM* and *HdCUC3* were cloned into pGADT7 and pGBKT7, respectively. HdCUC3 was fused to the GAL4 DNA binding domain (BD) to generate the HdSTM-BD bait construct, and HdSTM was fused to the GAL4 activation domain to generate the prey construct. The constructs were confirmed by sequencing and transformed into yeast strain AH109. Protein interactions were examined as previously described (Zhao et al., 2018). The primers used for the yeast two-hybrid assays are detailed in Supplemental Table S2.

### BiFC assay

The full-length coding sequences of *HdSTM* and *HdCUC3* without the stop codons were amplified by PCR using gene-specific primers and introduced into the pSPYNE-35S and pSPYCE-35S vectors containing the N- or C-terminus of YFP, respectively, to construct in-frame fusion proteins (Walter et al., 2004). The two plasmids were transformed into *Agrobacterium* strain GV3101 and co-transformed into the abaxial sides of 5- to 6-week-old *N. benthamiana* leaves to examine protein interactions as previously described (Zhao et al., 2018). The YFP signals were detected 48 h after co-infiltration under a Leica SP8 confocal laser microscope with an excitation wavelength of 488 nm. Primer information is given in Supplemental Table S2.

### Accession numbers

Sequence data from this article can be found in the GenBank/EMBL data libraries under accession numbers *HdACT1* (MZ365289), *HdSTM* (MZ365290), and *HdCUC3* (MZ365291).

## Supplemental data

The following materials are available in the online version of this article.

**Supplemental Figure S1.** Multiple sequence alignment of HdSTM and its homologs.

**Supplemental Figure S2.** Phylogenetic analysis of HdSTM and its homologs.

**Supplemental Figure S3.** Relative expression of *HdSTM* in different organs of *H. difformis*.

**Supplemental Figure S4.** RNA in situ hybridization with sense probes of *HdSTM* and *HdCUC3* in terrestrial and submerged shoots.

**Supplemental Figure S5** Analysis of GUS expression driven by the upstream region of *HdSTM* and identification of the CNSs in transformed *A. thaliana*.

**Supplemental Figure S6.** Multiple sequence alignment of HdCUC3 and its homologs.

**Supplemental Figure S7.** Phylogenetic analysis of HdCUC3 and its homologs.

**Supplemental Figure S8.** Relative expression of *HdCUC3* in terrestrial and submerged shoots.

**Supplemental Table S1.** The 1.8-kb promoter sequence of *HdSTM* including the RB-box and K-box.

**Supplemental Table S2.** Primers used in this study.

**Supplemental Table S3.** The genes used in this study.

## Supplementary Material

kiac382_Supplementary_Data

## Notes

H.W.H., S.K., and G.J.L. conceived and designed the research. G.J.L., J.J.Y., and Y.M.C. conducted most of the experiments. X.Y.Z. and Y.C. helped with vector construction. S.Q.H. improved the manuscript. All authors read and approved the final manuscript.

The author responsible for distribution of materials integral to the findings presented in this article in accordance with the policy described in the Instructions for Authors (https://academic.oup.com/plphys/pages/general-instructions) is Hongwei Hou (houhw@ihb.ac.cn).
