# Supplementary material for: SHOOT MERISTEMLESS participates in the heterophylly of Hygrophila difformis (Acanthaceae)
Source: Plant Physiol. 2022 Aug 19;190(3):1777–91. doi: 10.1093/plphys/kiac382 (PMC9614456; doi:10.1093/plphys/kiac382)
Supplement: kiac382_Supplementary_Data [file kiac382_supplementary_data.pdf]

Supplemental Data

Supplemental Figures

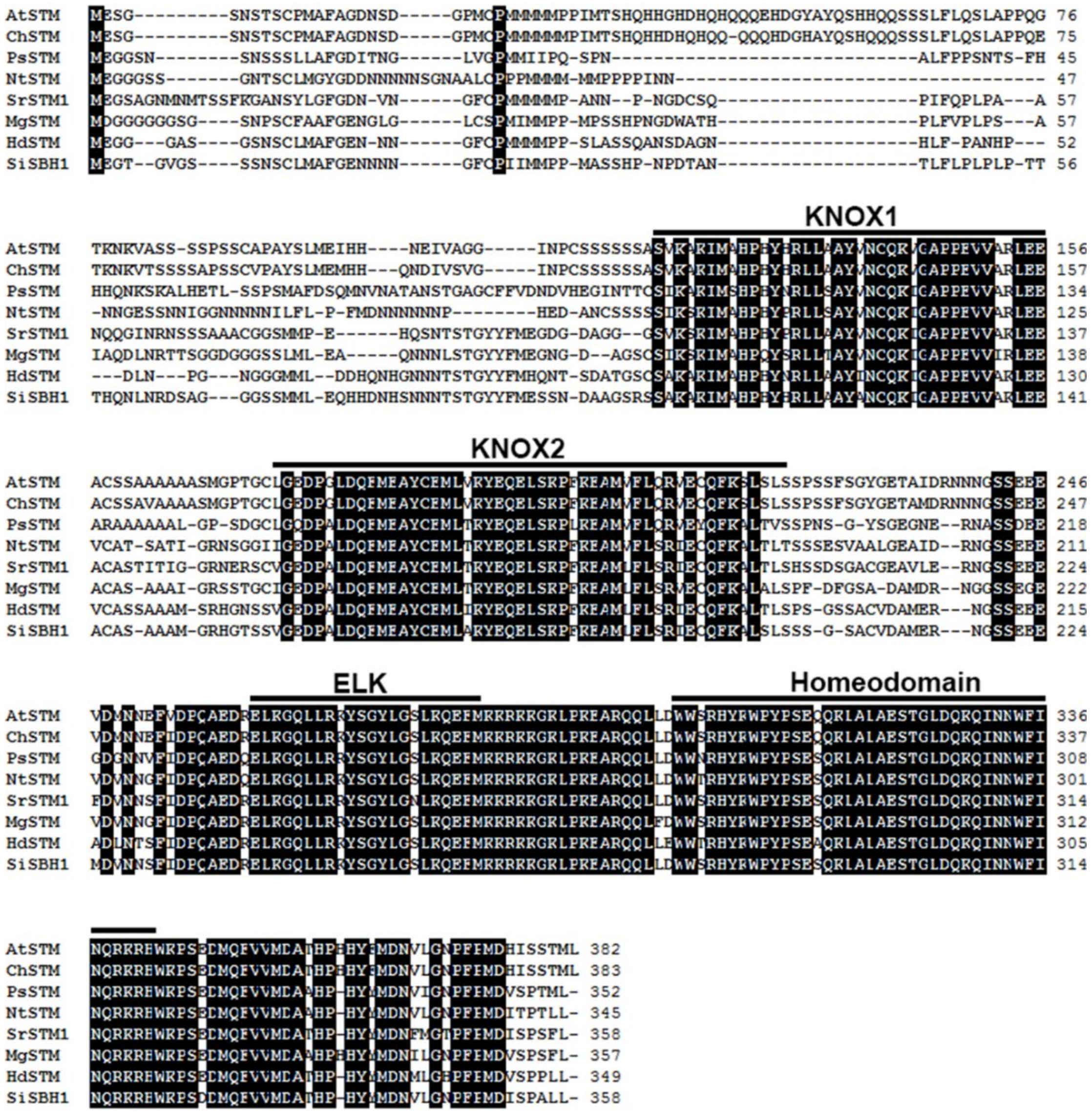

Supplemental Figure S1. Multiple sequence alignment of HdSTM and its homologs.

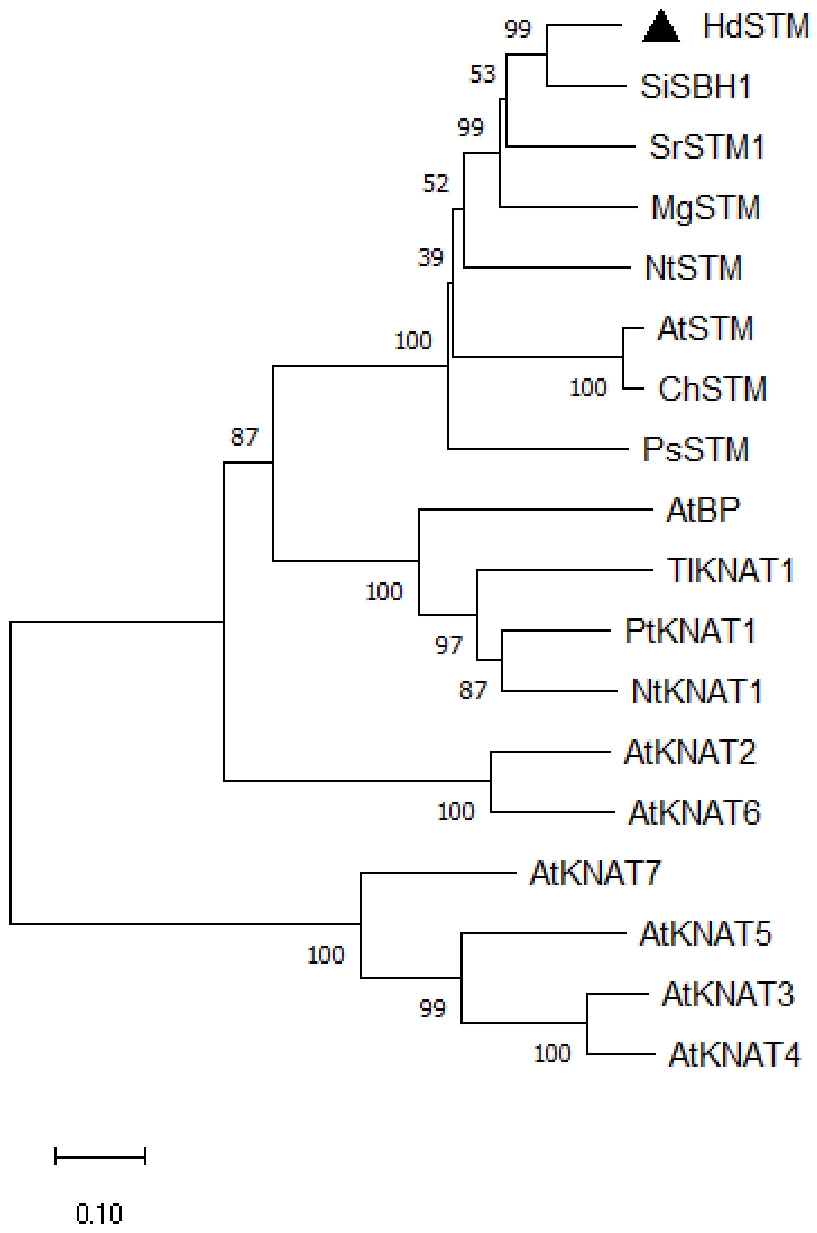

Supplemental Figure S2. Phylogenetic analysis of HdSTM and its homologs.

The numbers near the nodes indicate bootstrap value. Triangle represents protein sequence from *H. difformis*. Scale bar indicates average nucleotide substitutions per site.

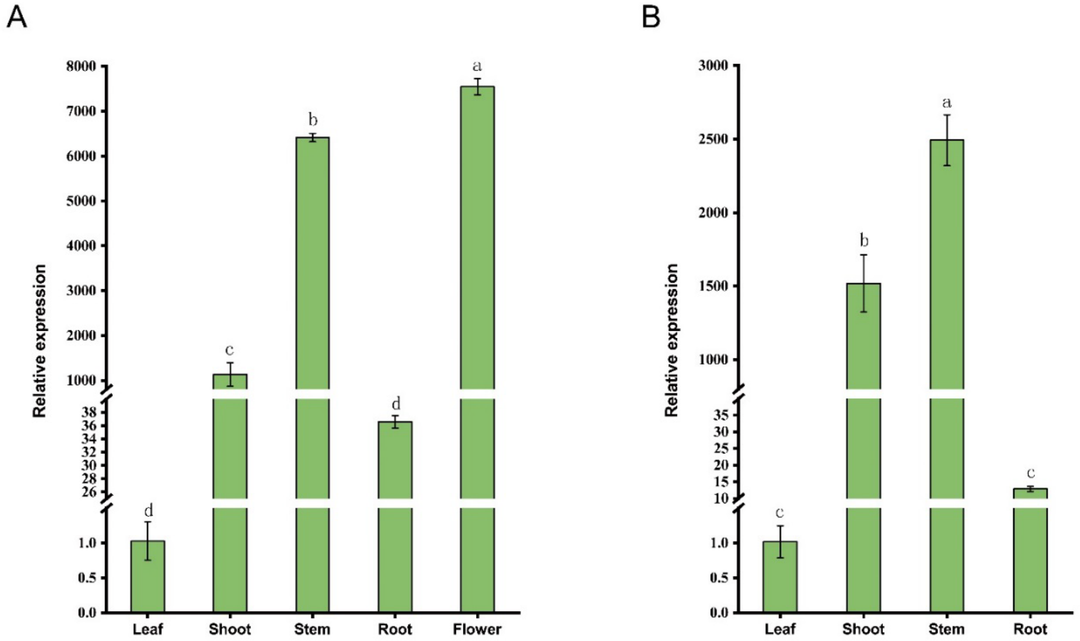

Supplemental Figure S3. Relative expression of *HdSTM* in different organs of *H. difformis*.
(A) Relative expression of *HdSTM* in terrestrial organs of *H. difformis*. (B) Relative expression of *HdSTM* in submerged organs of *H. difformis*. Three biological replicates were performed for each sample. Error bars represent ± SD. All expressions were normalized to mature leaf (at stage P6) in terrestrial or submerged conditions. Lowercase letters indicate significant differences determined by Tukey's test of one-way ANOVA ($P < 0.05$).

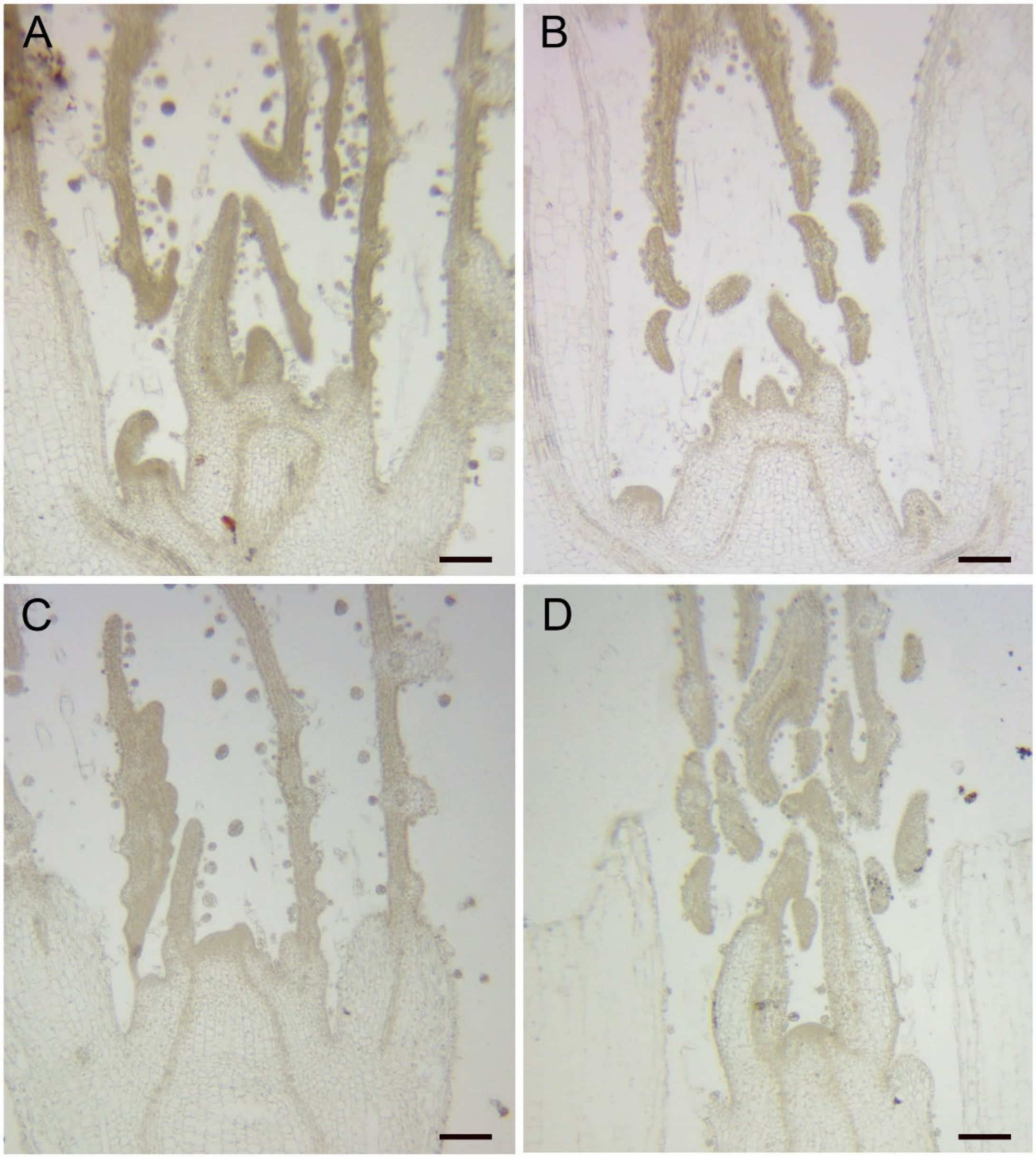

Supplemental Figure S4. RNA in situ hybridization with sense probes of *HdSTM* and *HdCUC3* in terrestrial and submerged shoots.

(A) RNA in situ hybridization of *HdSTM* in a terrestrial shoot. (B) RNA in situ hybridization of *HdSTM* in a submerged shoot. (C) RNA in situ hybridization of *HdCUC3* in a terrestrial shoot. (D) RNA in situ hybridization of *HdCUC3* in a submerged shoot. Bars = 0.5 mm.

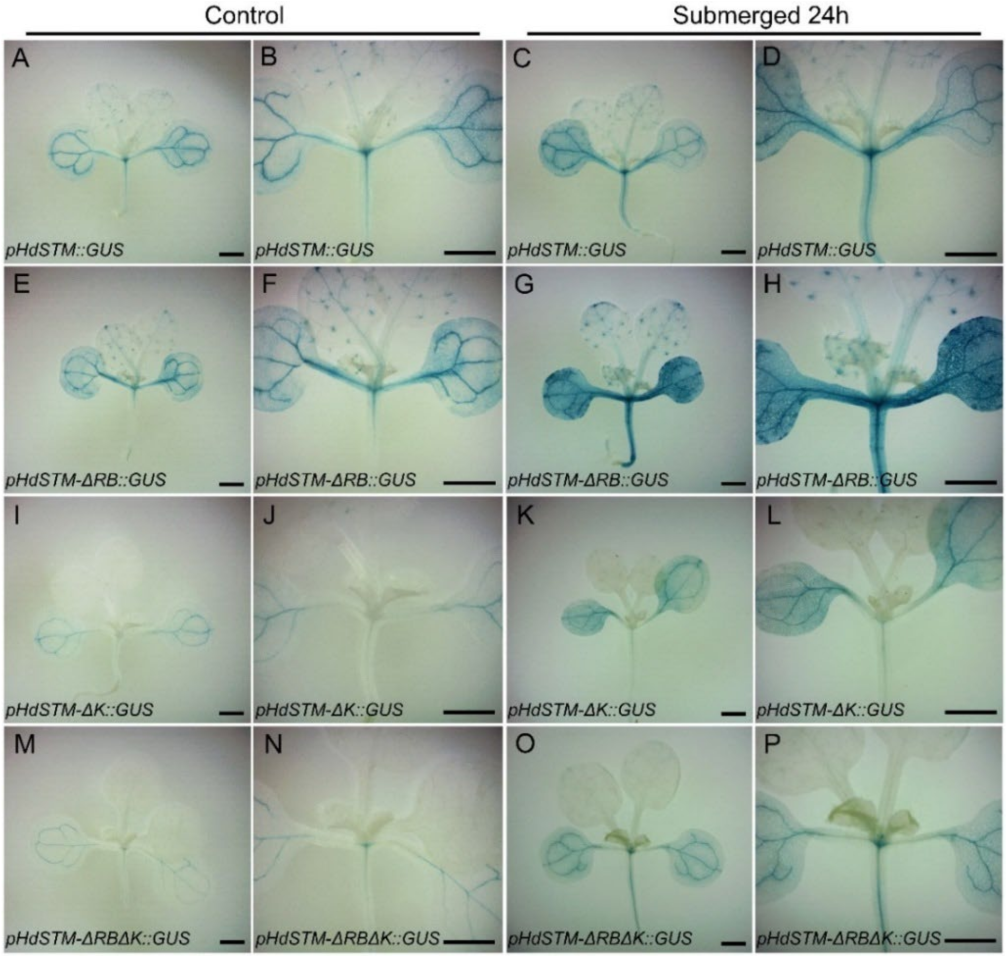

Supplemental Figure S5. Analysis of GUS expression driven by the upstream region of *HdSTM* and identification of the conserved noncoding sequences (CNSs) in transformed *A. thaliana*.

(A) GUS expression in *pHdSTM::GUS* transgenic *A. thaliana* seedlings; a magnified view is shown in (B). (C) GUS expression in *pHdSTM::GUS* transgenic *A. thaliana* seedlings after 24 h of submergence; a magnified view is shown in (D). (E) GUS expression in *pHdSTM-ΔRB::GUS* transgenic *A. thaliana* seedlings; a magnified view is shown in (F). (G) GUS expression in *pHdSTM-ΔRB::GUS* transgenic *A. thaliana* seedlings after 24 h of submergence; a magnified view is shown in (H). (I) GUS expression in *pHdSTM-ΔK::GUS* transgenic *A. thaliana* seedlings; a magnified view is shown in a (J). (K) GUS expression in *pHdSTM-ΔK::GUS* transgenic *A. thaliana* seedlings after 24 h of submergence; a magnified view is shown in (L). (M) GUS expression in *pHdSTM-ΔRBΔK::GUS* transgenic *A. thaliana* seedlings; a magnified view is shown in (N). (O) GUS expression in *pHdSTM-ΔRBΔK::GUS* transgenic *A. thaliana* seedlings after 24 of submergence; a magnified view is shown in (P). Pictures shown here are representatives of at least twenty individuals. Bars = 1 cm.

```
PsCUC3  ---------------------MMLAMEEVLCELSDHEKKNDQ----GLPPGFRFHPTDEELITFYLASKVFKNTFFN--------NVKFA 57
HdCUC3  MHSSHHHSFISRFAFRSVRNMMMVGIEDILCELDDDEMVNEKGGGVGLPPGFRFHPTDEELLTYYLASKVFHDTFSNNSSSSATALIHIA 90
AtCUC3  ---------------------MMLAVEDVLSEL-AGEERNER----GLPPGFRFHPTDEELITFYLASKIFHGGLS---------GIHIS 55
ChCUC3  ---------------------MMLAVEDVLSEL-AGEERNER----GLPPGFRFHPTDEELISFYLASKVFDGGLC---------GIHIT 55
AcCUC3  --------------------MMMFGMEEVLCEL-HGEDTNEQ----GLPPGFRFHPTDEELITFYLASKVFNGRFC---------GVEIA 56
StCUC3  ----------------------MLAMEEILCELNREEMMNEQ----GLPPGFRFHPTDEELITFYLASKVFNATFSA--------GIQIP 56
ZtCUC3  ----------------------MFPVEELLAEF-AGEDPADR----GLPPGFRFHPTDEELITFYLASKVFNGSFC---------GVEIA 54
CpCUC3  ----------------------MLAVEDVLRELGVGEEVNEQ----GLPPGFRFHPTDEELITFYLASKVLNATFS---------GLHIA 55

PsCUC3  EVDLNRCEFWELPDMAKMGEREWYLFSLRDRKYPTGLRTNFATCAGYWRATGRDKEVYSNNST-----RALLGMKRTIVFYKGFAPRGEK 142
HdCUC3  QIDLNRCEFWDLPDVAKIGERKWYFFSLRDRKYPTGLRTNFATCAGYWRATGRDREIYSSTSGGGGGRRLLLGMKRTIVFYKGFAPRGQK 180
AtCUC3  EVDLNRCEFWELPEMAKMGEREWYFYSLRDRKYPTGLRTNFATTAGYWRATGRDKEVFSGGGG------QLVGMKRTIVFYKGFAPRGLK 139
ChCUC3  EVDLNRCEFWELPEMAKMGEKEWYFYSLRDRKYPTGLRTNFATTAGYWRATGRDKEVFGSGGG------QLVGMKRTIVFYKGFAPRGLK 139
AcCUC3  EIDLNRCEFWELPDIAKMGERVWYFFSQRDRKYPTGLRTNFATEAGYWRATGRDKEVYSASDD------SLLGMKRTIVFYKGFAPRGVK 140
StCUC3  QVDLNRCEFWELPEVAKMGEREWYFFSLRDRKYPTGLRTNFATCAGYWRATGRDREVYSATNG------ALLGMKRTIVFYKGFAPRGEK 140
ZtCUC3  ELDLNRCEFWDLPDVAKMGEREWYFFSLRDRKYPTGLRTNFATCAGYWRATGRDREVYGATTG------VLLGMKRTIVFYRGFAPRGEK 138
CpCUC3  ELDLNRCEFWELPDVAKMGEKEWYFFSLRDRKYPTGLRTNFATCAGYWRATGRDREVYSASTG------ALLGMKRTIVFYKGFAPRGEK 139

PsCUC3  TFWVMHEYRLHTHLS--PSTCKEEWVICRIFHKSVEKRSSLLLQVQGHLG-VHNNSNLTPQKSCLPPPLPPPSFTHSHNNFPLHAFQPS- 228
HdCUC3  TFWVMHEYRLDGDFS-CRHSAKEEWVICRIFHKLGEKKGAANSMHQQQSYSYSMPKSAIPYAFNSLPPLLEYQSSKQ-DYTP-LMQQTLT 267
AtCUC3  TFWVMHEYRLENDHS-HRHTCKEEWVICRVFNKTGDRKNVGLIHNQISYLHNHSLSTTHHHHHEALPLLIEPSNKTL-TNFPSLLYDD-- 225
ChCUC3  TFWVMHEYRLETDLS-HRHSCKEEWVICRVFNKTGDRKNVGV-HSQISCLHNHSLSTYHHHHHETLPPLLEPS-KTI-SNFPSLLYDD-- 223
AcCUC3  TFWVMHEYRLEGDFSSFSHTFKEEWVLCRILQKTGEKKSPLFHAHNCFQEV-------FSSPSNFLPPMLEPSTTVT------------- 210
StCUC3  TFWVMHEYRLDGDFS-YRYSCKEEWVICRILEKVGEKKNAIYEGAGGGST--------YPTLKTWSSSSAAVNSPLT--------TNP-- 211
ZtCUC3  TFWVMHEYRLDGDFS-YRYSCKEEWVICRIFHKGGEKKNNSSFRQDVPVE--------D-----LPPTM----------------PP-- 195
CpCUC3  TFWVMHEYRLDGDFS-FRHTCKEEWVICRIFHKTGEKKNSVAAAAAASVL--------SPSRSSLAPVLLENQTPLL---------ES-- 209

PsCUC3  ----------FQV-------TDT-------------------------------------RNNNNPSLELLFKSQTIP------------ 252
HdCUC3  QNLQIPTQELSSSSSSSSTSSYLKSLINPLVSSQCDIFPVNFSHHHQPTTSFTSYYTPMSFMS--HQDHLSFK--------ELNNAFVGK 347
AtCUC3  PHQNYNNNNFLHGSSG-HNIDELKALINPVVSQLNGIIFP-----------S--------GNNNNDEDDFDFNLG--------------- 280
ChCUC3  HTHQNHNNNLFHGSSGHHHIDELKALINPVVSQLNGIIFS-----------P--------GNNNNVDDEDDFNLG--------------- 279
AcCUC3  QTPLHSLQNSFALKQE----SDLKAILNPYAIQLHDFHTN-----------FQSTYSPTSIKDTSPLSSFMFKNSLSNQDSILKEPTVPK 285
StCUC3  KTLQISHNNLYLFQ------HHENDLLKSLFNVSQT-TNL-----------L-------PMN----NNNVAFSSTKRYKDNK-QEEDMTN 271
ZtCUC3  HNKLN-LPVPFHGG------DA-R-----------YFT-------------------------------------------G-DLNSVTK 222
CpCUC3  QTHQTSPGNPFVTN------HHLKTLINPLLSQSNALINQ-----------L-------SAATNNTNSSILFKSLLSHQQQQ-LPSTVTK 274

PsCUC3  --KTEAMFYEQYQPQSIEEAIN-------LRWNIDNN-NSNDFQNLSLPVEMDAELIAFSGAAATDDEFTST--PFINSRG-IVALDAP- 328
HdCUC3  QCKKENPF-DGTHFQ------------ENARYHNPFLYGLSMN-------ECDGEMLRIPSATVNVD-DGNNATAMDPAFG-HVMSTEP- 414
AtCUC3  -VKTEQSS---------N--GNEID--VRDYLENPLFQE-AS-----------YGLLGFSSSP----------------GPLHMLLDSPC 328
ChCUC3  -VKTEPFL---------NGGSNELD--VRDYLENPLFHE-VG-----------YGLLGVSSAP----------------GPLHMLLDSPC 329
AcCUC3  QFKRETNF-PHFQPF--DATFNCMDKVQQNLWQDPLSFEMDC-----------SSVLGFSADDVDTVHEMSTSVA-FNRTRFQMMLDPPP 360
StCUC3  KNSSNSVV-INQQICMKSSRG--------NYLSSPLCYNNNDQNAFVMNSDWSSSLAGMEEG-----------IIPCNYFNNVVMDNCP- 340
ZtCUC3  MCKTESIM-SKLFHSPPDHAGQPANYS--SSIESPAAFDANG-----------SG----------------FPLVQQQRLGFRIEQDHQY 282
CpCUC3  QCKTEATF-SHFQILPVDAKMNWMDKI--NHISSSPLFSDMD-----------SSMLGPQGAAVD---DTSTSIAFNTLANFHLMLDQPI 347

PsCUC3  ----MGIDSWPQAQLV----- 340
HdCUC3  -----DFRWW----------- 419
AtCUC3  PLGFQL--------------- 334
ChCUC3  PLGFQL--------------- 335
AcCUC3  PII------------------ 363
StCUC3  --IKIAAESWPLHL------- 352
ZtCUC3  PFRLLGGESWPMDLSSHFAAT 303
CpCUC3  T-LPPAAESWPSIP------- 360
```

Supplemental Figure S6. Multiple sequence alignment of HdCUC3 and its homologs.

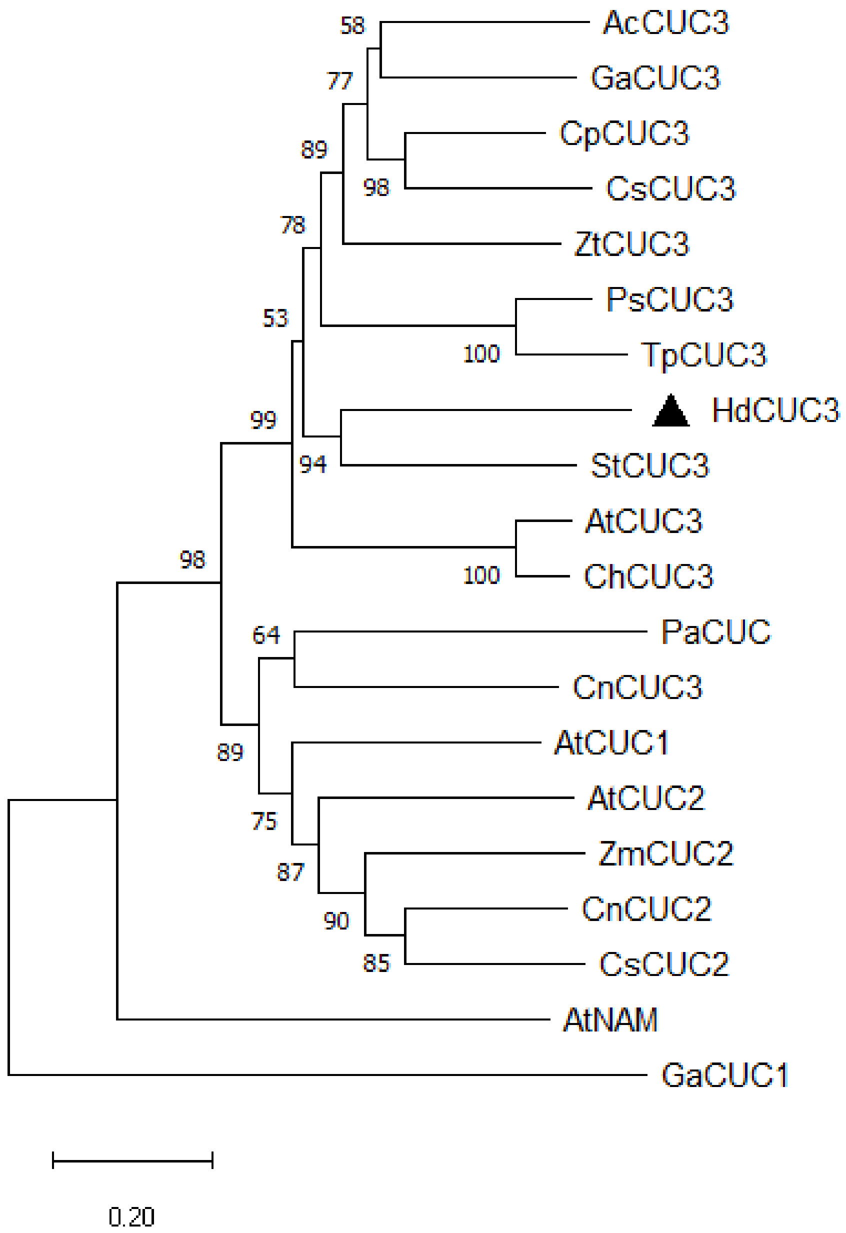

Supplemental Figure S7. Phylogenetic analysis of HdCUC3 and its homologs.

The numbers near the nodes indicate bootstrap value. Triangle represents protein sequence from *H. difformis*. Scale bar indicates average nucleotide substitutions per site.

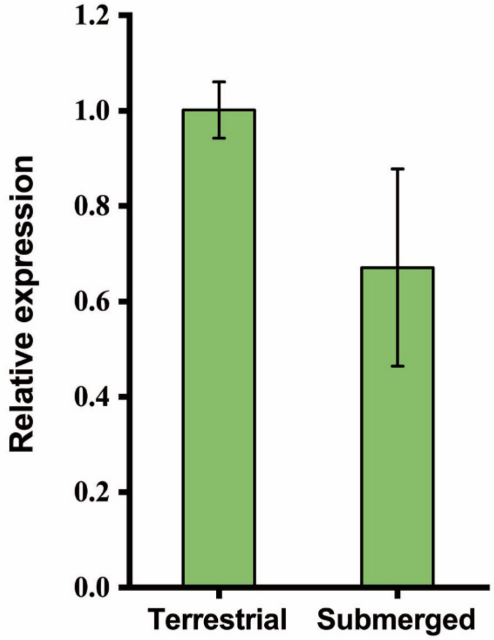

Supplemental Figure S8. Relative expression of *HdCUC3* in terrestrial and submerged shoots. Error bars represent ± SD (n=3). Statistical differences were determined using Student's *t*-test.

Supplemental Tables

Supplemental Table S1. The 1.8-kb promoter sequence of *HdSTM* including the RB-box and K-box.

GTCTTATTAGAATCGTATGATCACGTAGTTTTAGAATATACATTTTACATATTTTTTATTG
TGCATCTTTTGAGATATTAATGATAAAAATTGCATTGGATTGTGTGCAAAAGTCAAACA
AAACAAGTTATTGCATGGGATAGAGGGAGTACTTATAACTTACTAATACTCCCTCTGTT
TTCAAAAAGTTGCAACATTAATTCATTTCACGTAATCCAATACACATTCTTAACGCTTA
CTATCTCAAATTGTGTAAAAAGTAAAAATTATCAAAATTTGATATTTTAAAACTATGCG
ATTAGACGAATCGAACAAGATATCCTTTGACTATATTTTATGTTTTATAATTATTAAAAA
ATTGAATCAAAATTACTCAATAAATAATAATAACTACTATGCAAATGTTAAATTTATTAT
TAAAAACGGAGGGAGTATAATGGACTCGTAAGGCCTGCAACAAAAAAATCACATCCA
AAAATTACGAGTGAAATTAATGAAAGGGGAAAGAAAAGGGCAAAACAAATTTTAGG
GTTTTGAGAATGTGGCAAGGGTAAAGAGGCAGAAGAGGATAACACAGTAGCGAATA
ATAGGAGTGGACAAAAAAGCTCAATATCTATCTAGGCCACTAAAAGCATTCGTAAACA
CTCCACGGCCCTGTGATCCAAAATCGATCCCCGCCGTCAATCACAACTGGACGGCGC
AGATTGCTCCAAAACCCTAGGTTCGAAAAACCAGCACAGAAGCGGTGGTTCCGTGCC
CGAAAAACCAAACCTCCTCCGATAGTCCGATCCATTCATCACTAGCTAGCTGAGCTCC
CTCTCGCACAGTCACAGATAACACTCCAAACCCACACCTCGATTTGATCGTTCAATCA
CTCGTTTTCTAAAGGTTTGATTTTTTTTCCTTCTTTTTAAAAATTTATAAAGAAAAGCC
CACATTTCTCTCTCTCGTGTGCCAGTAATTGGCAGTGATTCAGTGGCAGTGCAAGGAA
AGAGAGCGAGAGTTTTCATTGGGAAGGTAAAAGCGTTGCTCTAAGAAAACATACATG
GGGTGGTTCCCTTGTGTCATCATCGTCATCAGCATCATCTCCTCTCTTGCCTAGAACCT
CAAATCCTATTTGTTACTACTCCTATTTCTCTCTCATCTGCAAAAAATCTCTCCTTTTCC
TTCACTGCAAATTTCTTTCTTTCAATCGTTTTTGTTTGTGTCTGTTCAGAGGGAGAGAA
TTTAATGCGTGTTAATGACCTGAGAAGTAAGAAAAACAACCAGAGTTTACACATTTCC
AATGGCAAAAAAAGAGTATATTTTTTTAAAAAAATTATAAAGGAACCTGAACAGGTTA
CTTTCTCAGAGACCATATCAAAGGCCAAGGGCAAAACCGCAAAACAACCCCGCCTTT
TCCATTCATCCATCTGACTACAGACTATATACTACCACCACTAACTACAACTACTATTTA
GGATTTCCTGTAAAAAGCCTGAATCAGTAAGCATAAAACTCGGGAGGCCCCTGCATA
GGTTCCATTCTCAAAGGGAAAAAAACAAATTTGAAGGAAAAAAGAGGAAAAAAAA
AAAAAAAAACAAAAAAGAAAAGAAAAGAAAAAGAAAGTGGAGCGAGGGATTGCT
TATGCGTGTGTACAACAGGAAAGAGAGTGCATAATGTTCTTCAAGGGTTTATAAACTG
AACTCTTTAAGGAGGTATTCTGATTACCGGAGATTGCAGAGGGGTTGTTGCAGCAAGT
GCAACGACACGCATACTACGCAGCAGGAGAAAAGCAGAGACGAAAAGCAAGCCTAA
Aatg

Note: RB-box, green; K-box, blue; Start codon, red.

Supplemental Table S2. Primers used in this study.

Primers used for gene amplification and vector construction	
HdSTM-clone-F	ATGGAAGGTGGTGGTGCCTCTGGAAGCA
HdSTM-clone-R	CCGATGGCACTAAATTCGTTTTCTCA
HdCUC3-clone-F	TTCTATCTGTCTTAGCTCTGTGTTGTGCA
HdCUC3-clone-R	AGGGAACTTGGGATTTTGAATATATGGT
HdSTM-in situ-F	AAGCCATGCTTTTCCTTTC
HdSTM-in situ-R	ATCAGCCTCTTCCTCGGAC
HdCUC3-in situ-F	CTCAAATCGACCTCAACCG
HdCUC3-in situ-R	CCGGTTCGTCCTCAGCCC
HdSTM-OE-F	ATGGAAGGTGGTGGTGCCTCT
HdSTM-OE-R	AAGAAGAGGAGGAGAGACGTCC
HdCUC3-OE-F	ATGCATTCTTCTCATCATCATTCA
HdCUC3-OE-R	CCACCACCTGAAATCCG
HdSTM-Sense-F	AAGGTGGTGGTGCCTCTGG
HdSTM-Sense-R	CTGCCCGTAGCATCACTGG
HdSTM-Antisense-F	AAGGTGGTGGTGCCTCTGG
HdSTM-Antisense-R	CTGCCCGTAGCATCACTGG
HdSTM-GFP-F	ATGGAAGGTGGTGGTGCCTCT
HdSTM-GFP-R	AAGAAGAGGAGTAGAGACGTCC
pHdSTM-GUS-F	GTCTTATTAGAATCGTATGATCA
pHdSTM-GUS-R	TTTAGGCTTGCTTTTCGTCTCT
pHdSTM-ΔRB-F	TCTCTCTCTCGTCTAAGAAAACATACATGGGGTGGTT
pHdSTM-ΔRB-R	TATGTTTTCTTAGACGAGAGAGAGAAATGTGGGCTTTTC
pHdSTM-ΔK-F	CTTTTCCATTCATCCATGCATAGGTTCCATTCTCAAAGG
pHdSTM-ΔK-R	AGAATGGAACCTATGCATGGATGAATGGAAAAGGCG
Primers used for RT-qPCR	
AtACT2-Q-F	TGGGATGAACCAGAAGGATG
AtACT2-Q-R	AAGAATACCTCTCTTGGATTGTGC
AtCUC1-Q-F	TTTGGTCAGTTTCTGGATTTG
AtCUC1-Q-R	CTCTAGAGCGGCCGCTCAGAGAGTAAACG

AtCUC2-Q-F	TAGCACCAACACAACCGTCACA
AtCUC2-Q-R	AGTTAACGTCTAAGCCCAAGGC
AtCUC3-Q-F	CTCAAGACTAAGTGGGTCATGC
AtCUC3-Q-R	CACTCTGCAAATCACCCATTCC
HdACT1-Q-F	GCCCTGAGGTCCTGTTCCA
HdACT1-Q-R	GCTCATCCTGTCGGCAATA
HdSTM-Q-F	CCCCATCTGGTTCTTCTGC
HdSTM-Q-R	TTCCCTGTCCTCGGCTTG
HdCUC3-Q-F	GGGAAAGACAGGGAGATTTACAGT
HdCUC3-Q-R	TGGCGGCAAGAGAAGTCACC
Primers used for yeast two-hybrid and BiFC assays	
HdSTM-Y2H-F	TAATACGACTCACTATAGGG
HdSTM-Y2H-R	AGATGGTGCACGATGCACAG
HdCUC3-Y2H-F	TAATACGACTCACTATAGGG
HdCUC3-Y2H-R	TTTTCGTTTTAAAACCTAAGAGTC
HdSTM-BiFC-F	ATGGAAGGTGGTGGTGCC
HdSTM-BiFC-R	AAGAAGAGGAGGAGAGACGTC
HdCUC3-BiFC-F	ATGCATTCTTCTCATCATCA
HdCUC3-BiFC-R	CCACCACCTGAAATCCGG

Supplemental Table S3. The genes used in this study.

Gene name	Species	Gene or protein ID in TAIR/GenBank
AtACT2	*Arabidopsis thaliana*	AT3G18780
AtCUC1	*Arabidopsis thaliana*	AT3G15170
AtCUC2	*Arabidopsis thaliana*	AT5G53950
AtCUC3	*Arabidopsis thaliana*	AT1G76420
HdACT1	*Hygrophila difformis*	MZ365289
HdSTM	*Hygrophila difformis*	MZ365290
AtSTM	*Arabidopsis thaliana*	AT1G62360
AtBP	*Arabidopsis thaliana*	AT4G08150
AtKNAT2	*Arabidopsis thaliana*	AT1G70510
AtKNAT3	*Arabidopsis thaliana*	AT5G25220
AtKNAT4	*Arabidopsis thaliana*	AT5G11060
AtKNAT5	*Arabidopsis thaliana*	AT4G32040
AtKNAT6	*Arabidopsis thaliana*	AT1G23380
AtKNAT7	*Arabidopsis thaliana*	AT1G62990
NtKNAT1	*Nicotiana tabacum*	AFY06676.1
PtKNAT1	*Populus tomentosa*	AKB11693.1
TlKNAT1	*Tropaeolum longifolium*	QZU26862.1
NtSTM	*Nicotiana tabacum*	AFY06675.1
SiSBH1	*Sesamum indicum*	XP_011086207.1
SrSTM	*Streptocarpus rexii*	AAW33774.1
MgSTM	*Monophyllaea glabra*	BAW35400.1
ChSTM	*Cardamine hirsuta*	ABF59514.1
PsSTM	*Polypleurum stylosum*	BAJ10711.1
HdCUC3	*Hygrophila difformis*	MZ365291
PaCUC	*Picea abies*	ADQ47506.1
GaCUC1	*Gossypium arboreum*	KHG10244.1
GaCUC3	*Gossypium arboreum*	KHG00366.1
ZmCUC2	*Zea mays*	NP_001288411.1

CnCUC3	*Cocos nucifera*	KAG1364514.1
CnCUC2	*Cocos nucifera*	KAG1334994.1
AtNAM	*Arabidopsis thaliana*	AT1G52880
CsCUC2	*Citrus sinensis*	KAH9802463.1
CsCUC3	*Citrus sinensis*	KAH9694023.1
StCUC3	*Solanum tuberosum*	NP_001275002.1
ZtCUC3	*Zeylanidium tailichenoides*	BBH72734.1
AcCUC3	*Aquilegia caerulea*	ACL14364.1
ChCUC3	*Cardamine hirsuta*	ACL14365.1
TpCUC3	*Trifolium pratense*	XP_045806298.1
PsCUC3	*Pisum sativum*	ACL14366.1
CpCUC3	*Carica papaya*	DAA34940.1
